# S-adenosyl-methionine (SAM) alters the transcriptome and methylome and specifically blocks growth and invasiveness of liver cancer cells

**DOI:** 10.18632/oncotarget.22942

**Published:** 2017-12-05

**Authors:** Yan Wang, ZhongSheng Sun, Moshe Szyf

**Affiliations:** ^1^ CAS Key Laboratory of Mental Health, Institute of Psychology, Chinese Academy of Sciences, Beijing, China; ^2^ Department of Pharmacology and Therapeutics, McGill University Montreal, Quebec, Canada; ^3^ Beijing Institutes of Life Science, Chinese Academy of Sciences, Beijing, China

**Keywords:** epigenetic, S-adenosyl methionine, liver cancer, mRNA sequencing, capture bisulfite sequencing

## Abstract

S-adenosyl methionine (SAM) is a ubiquitous methyl donor that was reported to have chemo- protective activity against liver cancer, however the molecular footprint of SAM is unknown. We show here that SAM selectively inhibits growth, transformation and invasiveness of hepatocellular carcinoma cell lines but not normal primary liver cells. Analysis of the transcriptome of SAM treated and untreated liver cancer cell lines HepG2 and SKhep1 and primary liver cells reveals pathways involved in cancer and metastasis that are upregulated in cancer cells and are downregulated by SAM. Analysis of the methylome using bisulfite mapping of captured promoters and enhancers reveals that SAM hyper-methylates and downregulates genes in pathways of growth and metastasis that are upregulated in liver cancer cells. Depletion of two SAM downregulated genes *STMN1* and *TAF15* reduces cellular transformation and invasiveness, providing evidence that SAM targets are genes important for cancer growth and invasiveness. Taken together these data provide a molecular rationale for SAM as an anticancer agent.

## INTRODUCTION

Broad changes in DNA methylation are a hallmark of cancer and are hypothesized to play a role in cancer initiation, progression and metastasis [1, 2]. Changes in DNA methylation in cancer cells include both increase in DNA methylation in promoters of many genes as well as reduced methylation in repetitive sequences and promoters of genes [1].

Studies revealed that hypomethylation of promoters of genes that are important for cancer metastasis is a common feature of cancer [3, 4] i.e. *Heparanase*[5] *Mmp2*[6–8] and *uPA*[9]. Genome wide analyses of DNA methylation changes in promoters in liver cancer [10] revealed that a significant fraction of promoters were hypomethylated.

Liver hypertrophy or carcinogenesis could be precipitated by agents that deplete SAM such as ethionine [11], choline deficient diets [12], methyl deficient diets [13] or ethanol [14]. SAM protects against hepatocarcinogenesis initiated with 1,2-dimethylhydrazine and promoted by orotic acid [15, 16] and is used to treat alcoholic liver disease [17] and protect against inflammation induced colorectal cancer [18]. However, the mechanism responsible for the anticancer effects of SAM in liver cancer is unknown. Moreover, the adverse effects that SAM might have on normal cell epigenetic programs have not been thoroughly investigated.

SAM is a global methyl donor and is predicted to effect all methylation reactions in the cell including DNA methylation [19], it is anticipated therefore that SAM treatment would have a global impact on the methylome and transcriptome. However, the genome wide impact of SAM treatment and how it relates to the phenotypic changes induced by SAM is unclear since most of the current reported data is anecdotal and limited to few candidate genes [20, 21]. A potential concern is that SAM might cause hypermethylation and downregulation of tumor suppressor genes and drive cancer growth. In addition, the effect that SAM might have on the methylome and transcriptome in normal cells is unknown; it is obviously critical to understand whether SAM exhibits any selectivity to cancer cells and cancer promoting genes.

SAM is an attractive anticancer agent since it is an approved nutritional supplement, however in absence of an understanding of its genomic targets and its selectivity to cancer cells, it is hard to make a case for its clinical use in treating or preventing HCC. We therefore characterized in this paper the effect of SAM on the methylome in primary liver cells and hepatocellular carcinoma cell lines. Our results identify particular gene pathways that are targeted by SAM. We show phenotypic as well as epigenomic and transcriptomic selectivity of SAM to cancer cells. Our data provide molecular support for the hypothesis that SAM is reversing cancer and metastasis related epigenomic programs and provide justification for further examining SAM as a candidate agent for preventing and reversing hepatocellular carcinoma.

## RESULTS

### SAM inhibits cell growth, invasiveness and anchorage independent growth selectively in cancer cells

**Figure 1:**
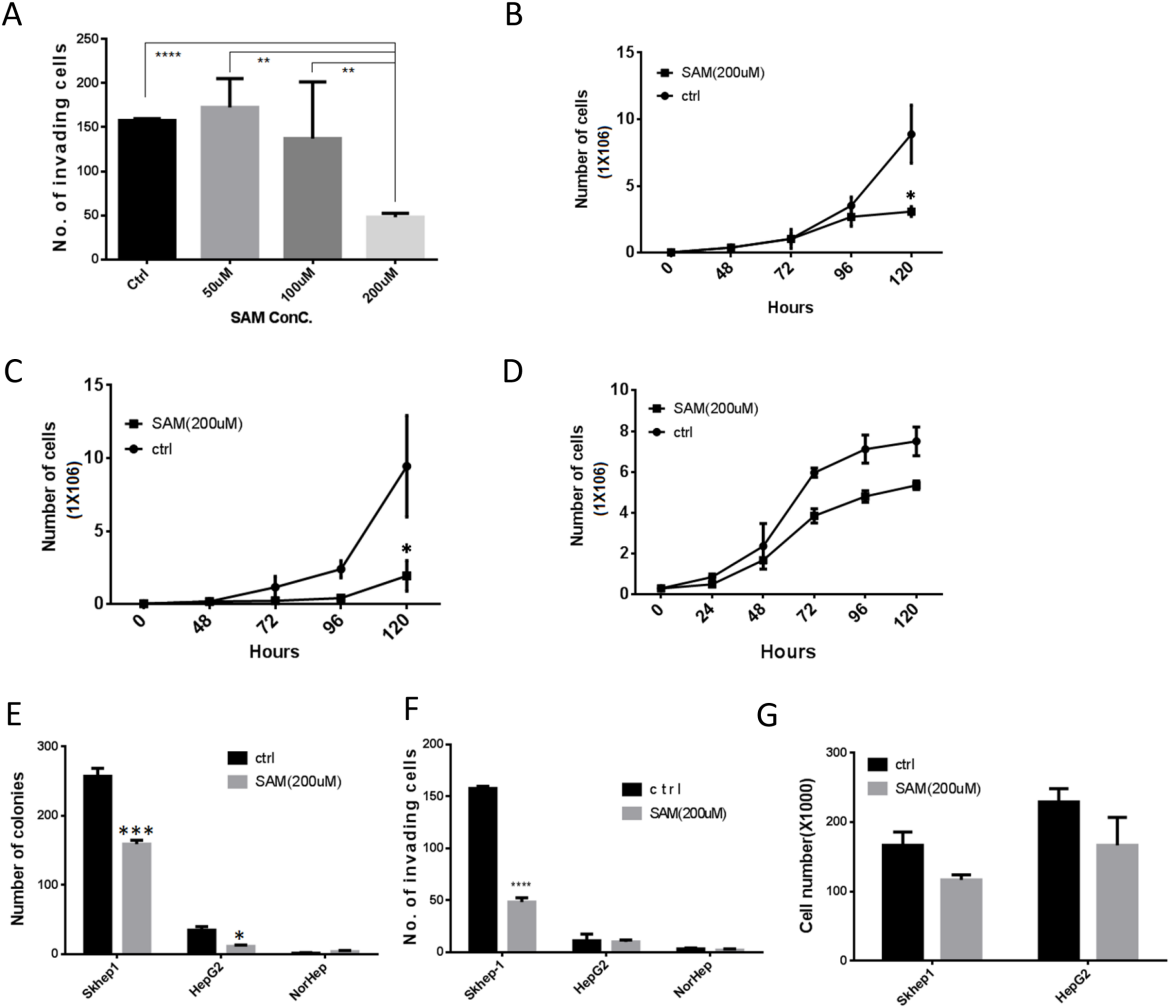
SAM decreases cancer cell growth and invasiveness selectively in liver cancer cells **(A)** Dose response of the effect of SAM on invasiveness of SKhep1 cells as measured by Boyden Chamber invasion assay. Invasion was quantified 24h after plating. **(B-D)** Time course of growth of HepG2 (B) SKhep1(C) and primary liver NorHep cell lines (D) in the presence of control medium and buffer (SAM dissolution buffer) or medium containing 200 μM of SAM. Cell growth was determined by counting live cell numbers at indicated time points using a Coulter counter. (**E)** Anchorage independent growth measured by soft-agar assay. **(F-G)** Effects of 200 μM of SAM on cell invasion (F) and viable cell counts 24h after plating under same conditions. (G) All results represent means ±SD of 3 determinations in either 2 or 3 independent experiments; ^****^, P<0.0001; ^***^, P < 0.001; ^**^, P < 0.01; ^*^, P < 0.05.

We first examined whether SAM selectively effects growth, transformation and invasiveness (a measure of metastatic potential) of liver cancer cells using two human liver cancer cell lines, HepG2 (HCC) and SKhep1 (liver adenocarcinoma) and primary untransformed liver cells (NorHep). SAM has a significant dose dependent effect on cell growth and anchorage independent growth (a measure of cellular transformation) (Supplementary Figure 1). We selected for further molecular experiments the dose of 200 μM since it also triggered a significant reduction of invasiveness in SKhep1 cells (Figure 1A), which is an important phenotype that we were interested in as an outcome measure of SAM treatment.

SAM inhibits growth of the two liver cancer cell lines HepG2 (Figure 1B) and SKhep1 (Figure 1C) but has a noticeably weaker effect at the same concentration on NorHep cells (Figure 1D). There was no noted cell death in either cell lines in response to SAM (Supplementary Figure 2). SAM treatment causes a significant reduction of anchorage-independent growth in SKhep1 and HepG2 (Figure 1E) but it doesn’t trigger anchorage independent growth in NorHep. SAM inhibits invasiveness of invasive SKhep1 cells but doesn’t induce invasiveness in HepG2 or NorHep (Figure 1F). In order to rule out the possible confounding anti-proliferative effects (Figure 1A, 1B) of SAM on cell invasion assays, we plated the same amount of cells onto a standard 6-well plate and counted viable cells 24hrs later concurrently with measuring invasiveness in the Boyden chamber assay. SAM treatment had no significant effect on cell numbers at this time frame suggesting that the observed anti-invasive effect of SAM is not caused by altered cell viability or proliferation rates (Figure 1G). In summary, SAM inhibits growth, anchorage independent growth and invasiveness of liver cancer cells selectively.

### Broad effects of SAM on the transcriptome in liver cancer and primary liver cells

**Figure 2:**
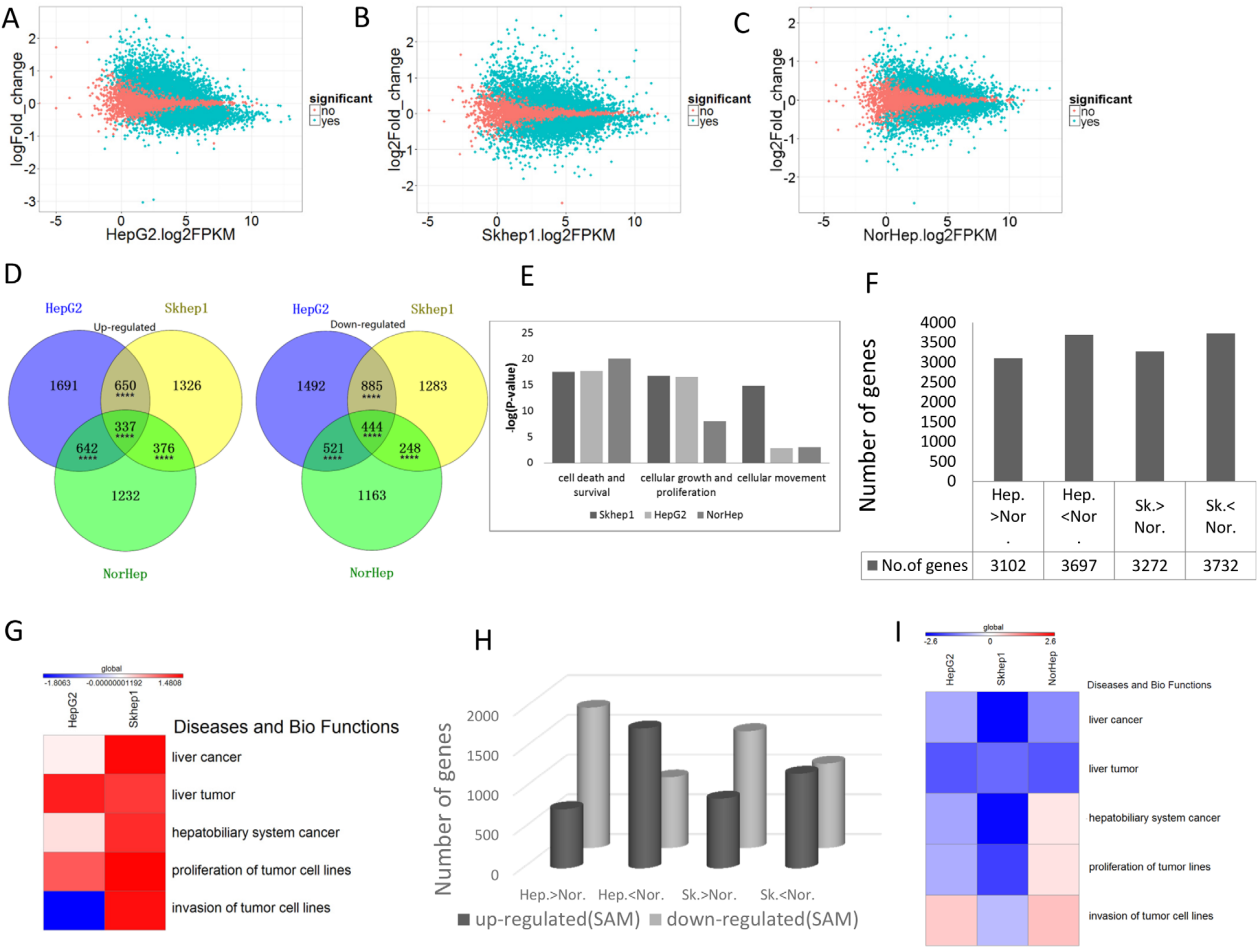
SAM causes broad cell-specific changes to the transcriptome mRNA from control and SAM treated cells was subjected to RNA sequencing and analysis as described in the methods. **(A-C)** MA-plot [M (log ratios); A (mean average)] of differentially expressed genes (DEGs) after SAM treatment of HepG2, SKhep1 and NorHep cell lines, respectively (q<0.05). DEGs are represented by green dots. Log2 fold change values for SAM treated vs. control samples are plotted against average log expression values (FPKM). **(D)** Venn diagrams of the significantly up-regulated and down-regulated genes among three cell lines in response to 200μM SAM treatment. **(E)** Chart of pathway categories associated with unique DEGs. The vertical axis represents the –log2 (P-value) of enrichment of the pathway in the respective cell line, and the horizontal axis represents the pathway category. **(F)** Chart of DEGs between untreated liver cancer cell lines and primary liver cells. **(G)** Heatmap of the z-scores of relative level of activation of pathways in categories related to diseases and biological functions enriched with DEGs between normal liver and cancer cell lines, a positive score indicates activated pathways relative to normal primary cells. **(H)** Number of DEGs between normal and cancer cell lines (Hep-HepG2; Nor-Normal; SK-SKhep1; the direction of difference in expression between untreated cell lines is indicated) whose expression is altered with SAM. **(I)** Heatmap of the z-scores of pathway categories related to diseases and biological functions enriched with genes whose expression is altered with SAM, a negative score (blue) indicates pathways that are downregulated with SAM treatment.

To understand the molecular mechanisms behind these effects of SAM and to delineate the potential functional consequences of SAM therapy on normal and cancer liver cells we performed mRNA sequencing of SAM treated and control HepG2, SKhep1 and NorHep cells. We used Cufflink (>2CPM; q<0.05) to delineate differentially expressed genes (DEGs) between cancer cell lines and NorHep and to determine the effect of SAM on mRNA expression. The differential expression of SAM/control (log2fold change) was plotted against baseline gene expression levels of the untreated control cells (log2FPKM) using ggplot2 in R [22] (Figure 2A-2C; significant changes between treated and controls marked in green). SAM caused broad changes in gene expression in both cancer and normal liver cells in both directions in all cell lines (3320 upregulated and 3342 downregulated in HepG2, 2860 upregulated and 2689 downregulated in SKhep1, and 2376 upregulated and 2587 downregulated in NorHep cell lines; Supplementary Table 4). Examination of the relationship between levels of gene expression in untreated cells and fold change in response to SAM (Figure 2A-2C) suggests that SAM downregulates genes that are highly expressed and upregulates genes that are poorly expressed in untreated cells (Supplementary Figure 3).

We examined how phenotypic differences between the effect of SAM on normal and cancer cell lines (Figure 1) might be explained by the differences in the effect on the transcription landscapes. The Venn diagram in Figure 2D reveals that although there is significant overlap between DEGs that are either upregulated or downregulated by SAM in the cancer lines and primary liver cells, there are many DEGs that don’t overlap, which might be behind the differences in the phenotypic responses (Figure 2D). We performed an IPA analysis on DEGs that were uniquely inhibited or activated by SAM in either the normal or one of the cancer liver cell lines. The top ten canonical pathways that were enriched with genes inhibited uniquely in SAM treated Skhep1cells were related to cancer, such as HIPPO signaling, ERK5 signaling, Rac signaling, while in HepG2, the top ranking pathway that was enriched with uniquely inhibited genes was the Acute Phase Response Signaling pathway. Role of BRAC1 in DNA Damage Response, ILK signaling and mTOR signaling pathways were enriched with genes uniquely activated by SAM in HepG2 cells but were enriched with genes inhibited by SAM only in Skhep1. This pathway may be related to cancer metastasis. Role of BRAC1 in DNA Damage Response pathways was also enriched by genes activated uniquely in NorHep DEGs. EIF2 signaling pathway which was reported to inhibit apoptosis was enriched by genes inhibited by SAM in all three cell lines consistent with the idea that SAM activates apoptosis (Supplementary Figure 4) [23].

Then we compared using IPA the significance of enrichment (Fisher’ exact test) of SAM treatment DEGs that are involved in the functional pathways of cell survival and death, cell growth and cellular movement and metastasis. Whereas the cellular death and survival pathway shows similar enrichment in SAM treatment DEGs in both NorHep and cancer cell lines, the proliferation pathway was highly enriched in HepG2 and SKhep1 relative to NorHep, while cell movement pathway was enriched in invasive SKhep1 cells (Figure 2E), which is consistent with the phenotypic effects of SAM on the three cell lines (Figure 1B-1D, 1G) [24].

### SAM reverses gene expression differences that differentiate liver cancer cells from normal primary liver cells

The IPA pathway analysis suggests that SAM targets different pathways in cancer and normal liver cells. We reasoned that SAM might act on genes that are differentially expressed between cancer and normal cells and reverse this difference, shifting the transcriptome of cancer cells “closer” to normal. 3102 genes are upregulated and 3697 genes are downregulated in HepG2 cells when compared with NorHep while 3272 genes are upregulated and 3732 genes are downregulated in SKhep1 in comparison with NorHep (Figure 2F). IPA analysis of DEGs between primary liver cells and SKhep1 and HepG2 using the Diseases and Biofunction category (heatmap in Figure 2G) reveals that several categories of liver disease are activated and enriched in both SKhep1 and in HepG2, while the invasiveness category is only enriched in invasive SKhep1 cells.

We then determined whether SAM treatment reverses the differences in enrichment in these pathways between cancer cell lines and normal cells. SAM treatment downregulates genes that are expressed at higher levels in cancer cells versus normal cells and upregulates genes that are expressed at a lower level in cancer cells (Figure 2H, Supplementary Table 5). IPA analysis using Diseases and Biofunction categories reveals that SAM reverses enrichment of DEGs (cancer over primary) in all liver cancer categories in both HepG2 and SKhep1 cells while the invasion category enrichment is downregulated only in the SKhep1 cells but invasion and proliferation categories are increased in NorHep cells (Figure 2I). Nevertheless, the activation of these pathways has no phenotypic effect in our assay in NorHep cells. These results demonstrate that SAM treatment acts on differentially expressed genes (cancer over primary) which are involved in cancer and invasiveness and reverses these differences (Supplementary Table 6). Gene set enrichment analysis (GSEAv2.2.3) revealed that genes whose expression increased after SAM treatment were significantly enriched within gene sets that were down-regulated in cancer vs. normal cells and genes whose expression decreased after SAM treatment were enriched within gene sets that were upregulated in cancer cells vs. normal cells (Supplementary Figure 5).

### SAM alters the methylation landscape of cancer and primary liver cells

SAM is the methyl donor of the DNA methylation reaction catalyzed by DNA methyltransferases and was previously reported to inhibit and reverse demethylation of several genes in cancer cells [25]. Changes in DNA methylation might be partly responsible for the changes in the transcription landscape triggered by SAM. We used the human SeqCap Epi CpGiant Enrichment Kit to map by bisulfite sequencing at single base resolution the promoters, exons and enhancer regions in untreated and treated NorHep, HepG2 and SKhep1 cells. 2,812,548 CpG positions were interrogated and 5,625,096 sites were captured on both strands. We obtained an average of 60 million reads per sample (Supplementary Table 7), 95% reads mapped to the human reference genome (hg19) using BSMAP. After deduplication, 60% of the reads were intersected to the target regions using bedtools2.24.0 [26] (Supplementary Figure 6A). We obtained more than 80% coverage of the target CpG sites represented on the arrays with average depth of coverage 18 (Supplementary Figure 6B-6C).

After extracting the methylation values, the distribution of CG methylation levels was plotted for the different samples before and after SAM treatment (histogram in Supplementary Figure 7A). As expected, the distribution was bimodal; a large fraction of CGs were either fully methylated or fully unmethylated. An examination of the different genomic features following annotation (vioplot; Supplementary Figure 7B-7D) showed a bimodal distribution across genomic features, however large differences were observed in the distribution of fully methylated versus unmethylated CGs in the different features. Promoters were enriched with fully unmethylated CGs, exons, introns 3’ and 5’ UTR were enriched with fully methylated CGs while intergenic regions showed distribution across the whole range of CG methylation states. Differences were noted between cancer cell lines and NorHep in each of the genomic features including promoters; a higher fraction of fully methylated promoters is observed in NorHep as compared to cancer cell lines. Median methylation level in promoters is 0 in HepG2, 0.018 in SKhep1 and 0.03 in NorHep. The mean methylation in each of the annotated elements is slightly higher in cancer cell lines treated with SAM but is lower in NorHep cell line treated with SAM relative to untreated cells (Supplementary Table 8).

**Figure 3:**
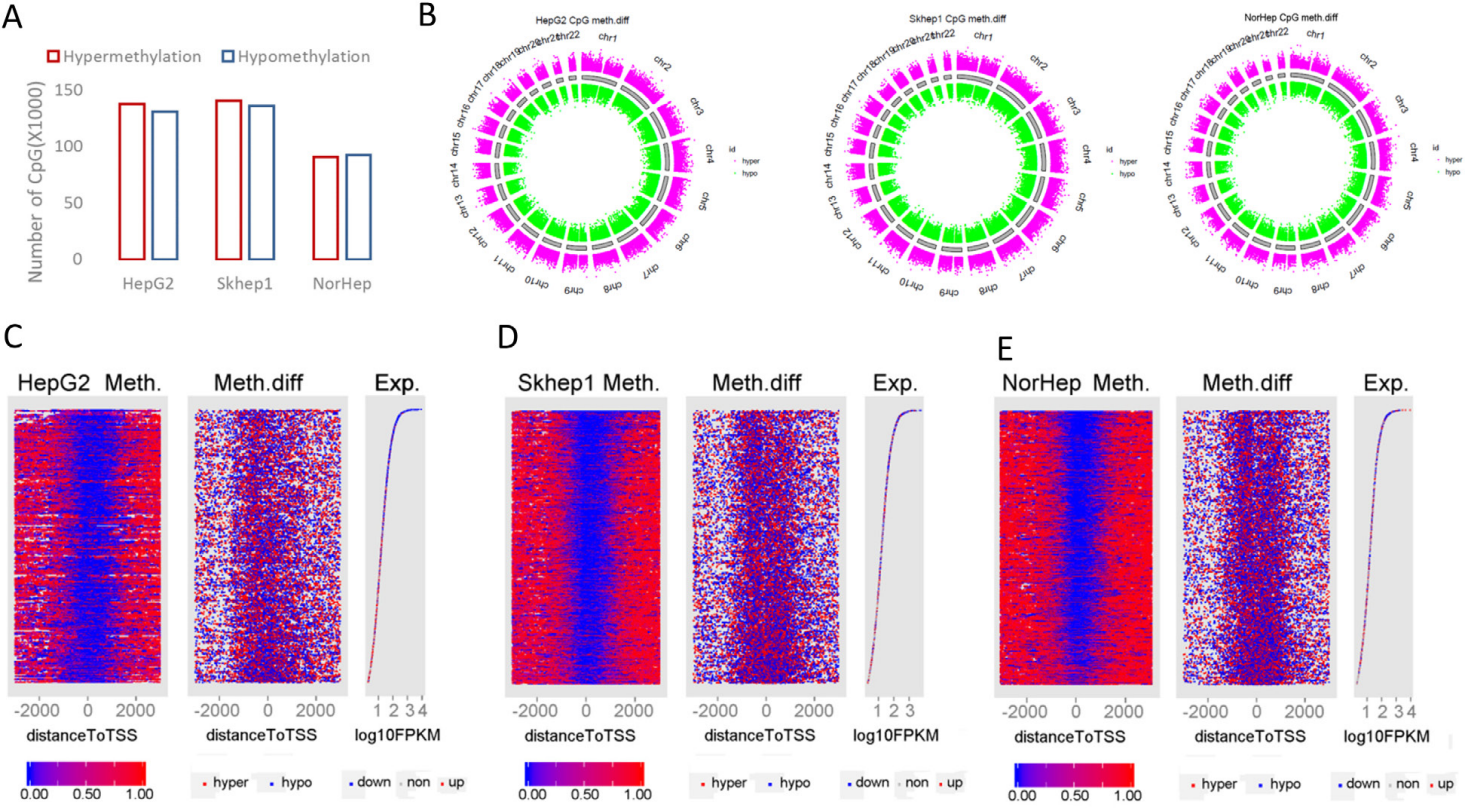
Genome wide response of the methylome to SAM treatment **(A)** Chart depicting the number of differentially methylated CpGs derived from capture bisulfite sequencing analysis computed by MethylKit package (q value<=0.05, %methylation difference >=15%). **(B)** Circular representation of the differentially methylated CpGs in all chromosomes (red: hypermethylation; green: hypomethylation relative to controls). **(C-E)** HepG2 (C), SKhep1 (D) and NorHep (E) cell lines. Left panels are heatmaps visualizing methylation levels of CpGs at TSS and flanking regions (±3kb) for all genes in untreated cells lined by the rank order of gene expression from high at top to low at the bottom, the profile of gene expression level for the corresponding genes is at the right; genes that were downregulated by SAM are represented in blue dots and genes upregulated by SAM are represented with red dots. The middle panel visualizes differentially methylated CGs after SAM treatment at TSS and flanking regions lined up by the same order as the right and left panels. Methylation level scales from 0% (blue) to 100% (red) are presented in the lower panel.

We noted both hypo- and hyper-methylation in response to SAM in all cell lines (Figure 3A). 52543, 29061 and 39679 nonCG methylation sites were hypomethylated, and 32545, 51868 and 36379 were hypermethylated in HepG2, SKhep1 and NorHep respectively (Supplementary Table 9). SAM doesn’t increase the average methylation of nonCG sites in either cancer cell lines. The differentially methylated CpGs (DMCs) were widely distributed across all chromosomes as shown in Figure 3B. Differentially methylated CGs are distributed across different genomic features with the largest number of differentially methylated CGs in promoters (Supplementary Figure 8). However, since the human SeqCap Epi CpGiant arrays are enriched for promoters and enhancers, the relative enrichment for differential methylation in promoters and H3k4me1 and H3k4me3 peaks was the lowest amongst genomic features (Supplementary Figure 9). Nevertheless, the fact that numerous SAM treatment DMCs are located in promoters and enhancers suggests that it is possible that SAM altered gene expression program through changes in DNA methylation of gene regulatory regions.

We tested the relationship between changes in DNA methylation and expression in response to SAM and the basal level of expression of these genes in untreated cells (Figure 3C-3E). The left panels (for each of C-E) represent the level of methylation of each CG site and its position relative to the TSS per gene. Genes are lined up from top to bottom by their levels of expression in untreated cells (plotted in the right panels of 3C-E), changes in expression after SAM treatment are indicated on the expression plot by color of the dots representing each gene (blue=downregulation with SAM; red=upregulation with SAM). The middle panels show changes in methylation for each CG site in response to SAM (blue=SAM<untreated; red=SAM>untreated). The distribution of CG methylation follows a common profile with high methylation on both edges of the TSS methylation-free zones. SAM introduces new methylation sites to the TSS proximity zones. Highly expressed genes in untreated cells are inhibited and differentially methylated in response to SAM (Figure 3C-3E, blue dots in right panels). We used gene set enrichment analysis (GSEAv2.2.3) to gain insight into the relationship between changes in methylation and gene expression in response to SAM treatment in the three cell lines. We defined gene sets that were enriched with hyper- and hypomethylated genes in response to SAM using the Cancer Module database. Leading edge analysis was performed on the significantly enriched gene sets (FDR<0.2 and NOM qval<0.05). The analysis revealed that the hypomethylated gene sets are enriched with gene subsets with increased expression and that the hypermethylated gene sets are enriched with downregulated gene subsets, which is consistent with the idea that changes in DNA methylation were accompanied by anticorrelated changes in gene expression (Supplementary Figure 10).

### SAM reverses differences in DNA methylation and expression profiles between liver cancer cells and normal primary liver cells

**Figure 4:**
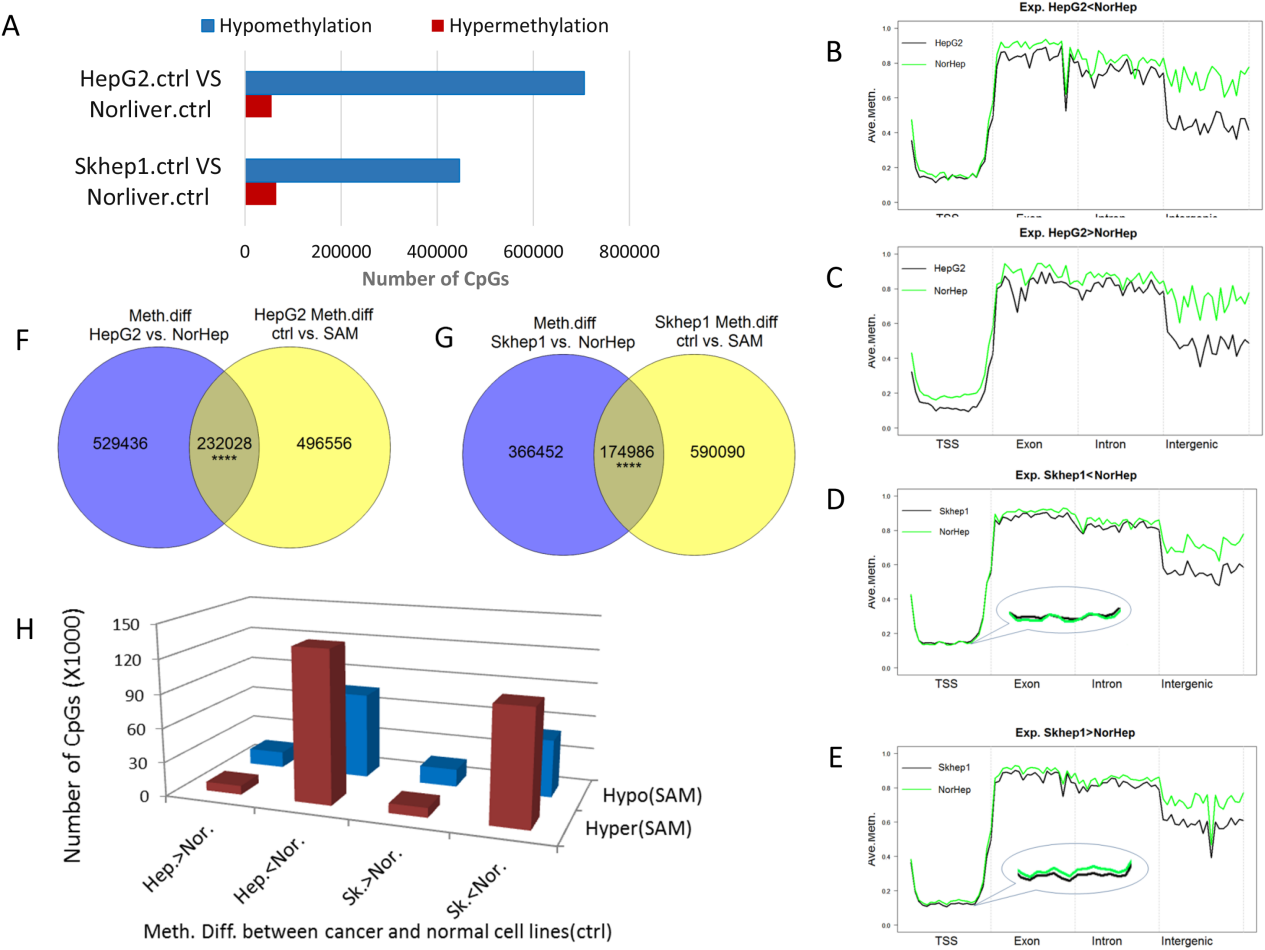
SAM reverses differences in DNA methylation and expression between liver cancer cells and normal primary liver cells **(A)** Chart depicting the number of differentially methylated sites between either SKhep1 or HepG2 and primary liver cells (NorHep) (q<0.05, %methylation difference>=20%). **(B-E)** A plot of methylation levels across composite gene features of genes that are either downregulated in HepG2 (B) or SKhep1 cells (D) or upregulated in HepG2 (C) or SKhep1 cells (E) relative to NorHep. DNA sequence is split into 20 windows of each annotated element along the gene. The y-axis shows the mean percentage methylation for each window. **(F-G)** Venn diagrams depicting the overlap of (differentially methylated CGs) DMCs between SAM treated cells and their respective untreated controls versus DMCs between untreated HepG2 or SKhep1 and NorHep. **(H)** Chart depicting the number of DMCs that are altered in response to SAM treatment in genes that are either upregulated or downregulated in the respective cancer cell lines HepG2 and SKhep1 in comparison with NorHep (Hep-HepG2; Nor-Normal; SK-SKhep1; the direction of difference in methylation between cell lines is indicated).

SAM reverses phenotypic differences between cancer cell lines and NorHep (Figure 1). We first delineated DNA methylation differences between cancer cell lines and NorHep cells (q value<0.05 delta beta>0.2) (Figure 4A); the majority of differentially methylated sites are hypomethylated in cancer cell lines and these hypomethylation events include promoters as well as other genomic features (Supplementary Figure 11).

We examined the genomic features of differentially methylated sites between cancer and NorHep cells (20 bins in each annotation feature) and examined their profiles in genes that are down regulated (Figure 4B, 4D) versus genes that are upregulated in cancer cells (Figure 4C, 4E). Hypomethylation in the body of the genes and intergenic regions occurs in both genes that are upregulated as well as genes that are downregulated in the cancer cell lines. However, hypomethylation of promoter regions in HepG2 and to a lesser extent in SKhep1 cells occurs only in genes that are overexpressed in these cancer cell lines relative to NorHep cells.

We then examined whether SAM treatment reversed the differences in DNA methylation (DMC) between cancer and NorHep cells. As shown in Figure 4F, 4G there is a highly significant (Hypergeometric p=0) overlap between DMCs that differentiate cancer cell lines from NorHep and SAM triggered DMCs (∼30% of DMCs between cancer and normal cells were affected by SAM: SAM reduced the differences in methylation in ∼65% of these DMCs) (Figure 4G). SAM also affected sites that are not different between HepG2, SKhep1 and NorHep cells. The majority of sites that were hypomethylated in Cancer versus primary cells became hypermethylated with SAM treatment while sites that were hypermethylated in cancer versus primary cells became hypomethylated (Figure 4H). However, although the reversal of methylation with SAM was highly significant, SAM had as expected some sites changed in the same direction and other sites that were not originally different between cancer and control were nevertheless altered by SAM treatment.

**Figure 5:**
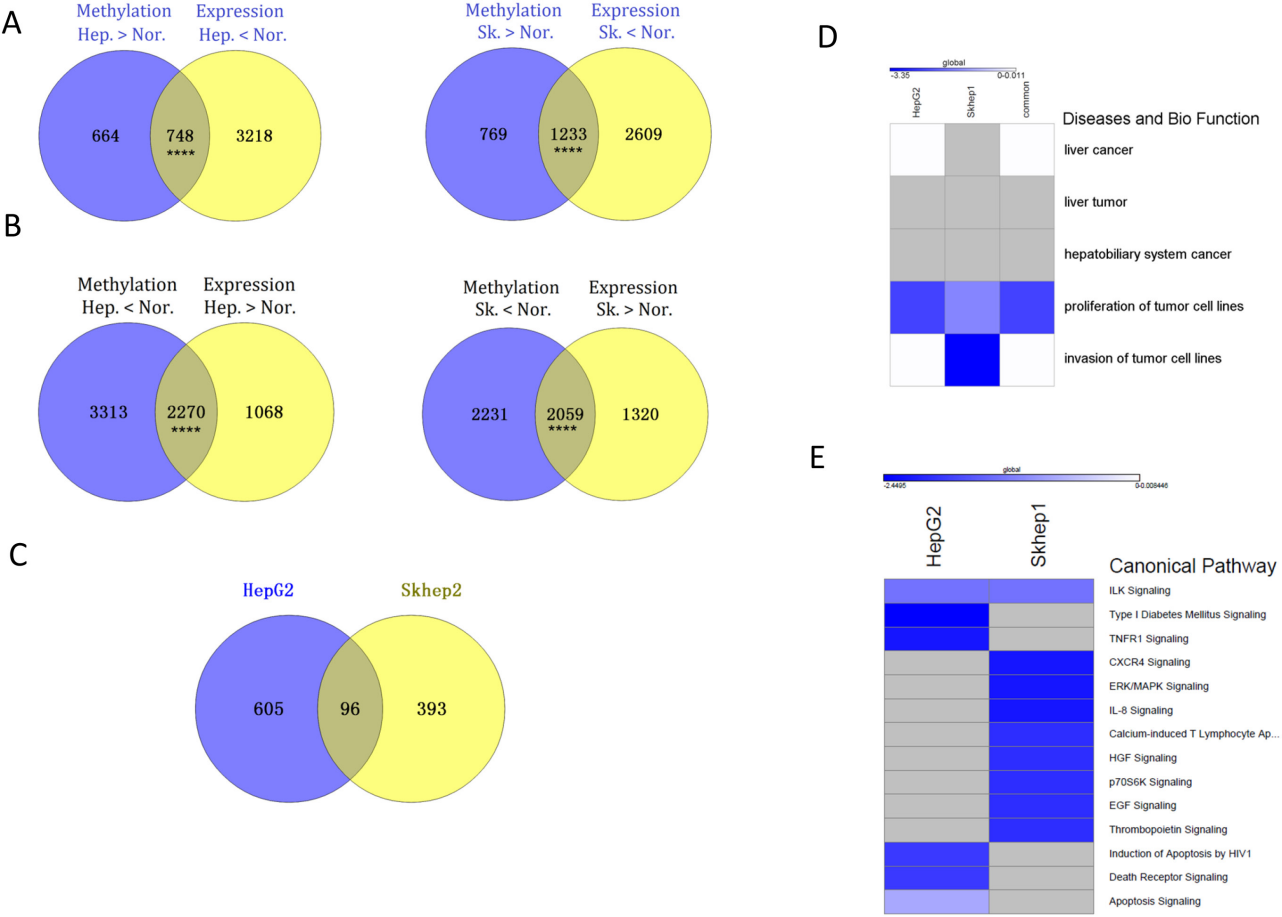
SAM hyper-methylates and down-regulates genes involved in cell proliferation and metastasis that are upregulated in liver cancer cell lines (HepG2 and SKhep1) relative to NorHep cells **(A)** Venn diagrams showing overlap between genes that are hypermethylated and those that are downregulated in HepG2 or SKhep1 relative to NorHep cells; genes downregulated and hypermethylated in liver cancer cell lines. **(B)** Venn diagrams showing overlap between genes that are hypomethylated and those that are upregulated in HepG2 or SKhep1 relative to NorHep cells; genes hypomethylated and upregulated in liver cancer cell lines. **(C)** Venn diagrams of the overlap between genes that are hypomethylated and upregulated relative to NorHep and become hypermethylated and down regulated in response to SAM in SKhep1 and HepG2 cells. **(D-E)** Heatmaps representing z-scores of pathways (diseases and biological function/canonical pathway) enriched with genes that were hypermethylated and down-regulated in response to SAM treatment.

We determined how reversal of differences in methylation in promoter regions between cancer and control cell lines following SAM treatment associated with reversal of gene expression differences. 748 genes in HepG2 cells and 1233 in SKhep1 cells are both hypermethylated and downregulated relative to NorHep (Supplementary Table 10) (Figure 5A). SAM treatment causes demethylation and upregulation of 372 genes in HepG2 and 539 in SKhep1 cells (Supplementary Figure 12A, 12B). A large fraction of these genes effected by SAM in cancer cell lines are genes that are downregulated and hypermethylated in the untreated cancer cell lines relative to NorHep (229 genes out of 372, 61% in HepG2; 266 out of 539, 49.3% in SKhep1) (Fisher p=0.00011 for HepG2 and p= 0.00012 for SKhep1). 2270 and 2059 genes are both hypomethylated and upregulated in HepG2 and in SKhep1 respectively relative to NorHep (Figure 5B). 1132 genes are both downregulated and hypermethylated in HepG2 and 917 genes in SKhep1 in response to SAM treatment. A large fraction of genes effected by SAM are genes that are also hypomethylated and upregulated in untreated cancer cell lines versus NorHep (701 in HepG2, 62% and 489 in SKhep1, 53%) (Fisher, p=2.2e-16 for HepG2 and p=2.9e-8) (Supplementary Table 11). Thus, a large fraction of the changes in both methylation and expression caused by SAM reverse differences in DNA methylation and gene expression that exist between cancer and NorHep cells.

We listed the genes that were both hypomethylated and upregulated in either HepG2, SKhep1 or both cell lines relative to NorHep and whose expression and state of methylation was reversed by SAM (Figure 5C the overlap in the ven diagram). The results of an IPA analysis presented in Figure 5D (Supplementary Table 12) show that while the cell proliferation pathway was downregulated by SAM in both cancer cell lines, invasiveness was downregulated only in SKhep1, which is consistent with the phenotypic effects of SAM in these cancer cell lines (Figure 1). Analysis of genes that were hypermethylated and silenced in each of these cancer cell lines by SAM reveals different canonical pathways related to cancer growth and metastasis (Figure 5E) (see Table 1).

**Table 1:** List of validated genes that are hypomethylated and upregulated in liver cancer cells as compared with primary liver cells and were downregulated by SAM (log2FC-log2 fold change)

Gene	Log_2_FC SAM/untreated	Description	Pathway
SKhep1 unique(Metastasis-related)			
DDIT3	-1.76	DNA-damage-inducible transcript 3	MAPK signaling pathway,
MIA	-1.17	Melanoma Inhibitory Activity	MIA pathway
TAF15	-1.08	TAF15 RNA polymerase II, TATA box binding protein (TBP)-associated factor	Assembly of RNA Polymerase II Complex; Estrogen Receptor Signaling
MTHF2	-0.80	methylenetetrahydrofolate dehydrogenase (NADP+ dependent) 2, methenyltetrahydrofolate cyclohydrolase	Glyoxylate and dicarboxylate metabolism, One carbon pool by folate,
ITGA6	-0.72	integrin, alpha 6	Focal adhesion, ECM-receptor interaction,……
NFIB	-0.72	nuclear factor I/B	
PBK	-0.67	PDZ binding kinase	
PDK1	-0.61	pyruvate dehydrogenase kinase, isozyme 1	T cell receptor signaling pathway, Fc epsilon RI signaling pathway, …..
CLIC4	-0.56	chloride intracellular channel 4	
SLC2A1	-0.40	solute carrier family 2 (facilitated glucose transporter), member 1	Pathways in cancer, Renal cell carcinoma,….
STMN1	-0.35	stathmin 1	MAPK signaling pathway,
SKhep1 and HepG2 common (proliferation related)			
NFIL3	-0.49	nuclear factor, interleukin 3 regulated	
RAN	-0.63	RAN, member of RAS oncogene family	Mechanism of Protein Import into the Sumoylation by RanBP2 Regulates Transcriptional Repression,……
TRIB3	-0.78	tribbles homolog 3 (Drosophila)	
PEG10	-0.46	Paternally Expressed 10	Validated targets of C-MYC transcriptional activation
CDT1	-0.53	chromatin licensing and DNA replication factor 1	CDK Regulation of DNA Replication,
DYNC1H1	-0.80	dynein, cytoplasmic 1, heavy chain 1	
RRM2	-0.72	ribonucleotide reductase M2 polypeptide	p53 signaling pathway,…
E2F1	-0.24	E2F transcription factor 1	Pathways in cancer, ….
HAT1	-0.45	histone acetyltransferase 1	Pathway member “HAT1”
CBS	-1.20	cystathionine-beta-synthase	Cysteine and methionine metabolism,…..
MYC	-0.73	v-myc myelocytomatosis viral oncogene homolog (avian)	MAPK signaling pathway, ErbB signaling pathway, …….
MCM3	-0.30	minichromosome maintenance complex component 3	DNA replication, Cell cycle

### Functional validation of genes that are hypomethylated and upregulated in liver cancer cells and are silenced by SAM treatment

**Figure 6:**
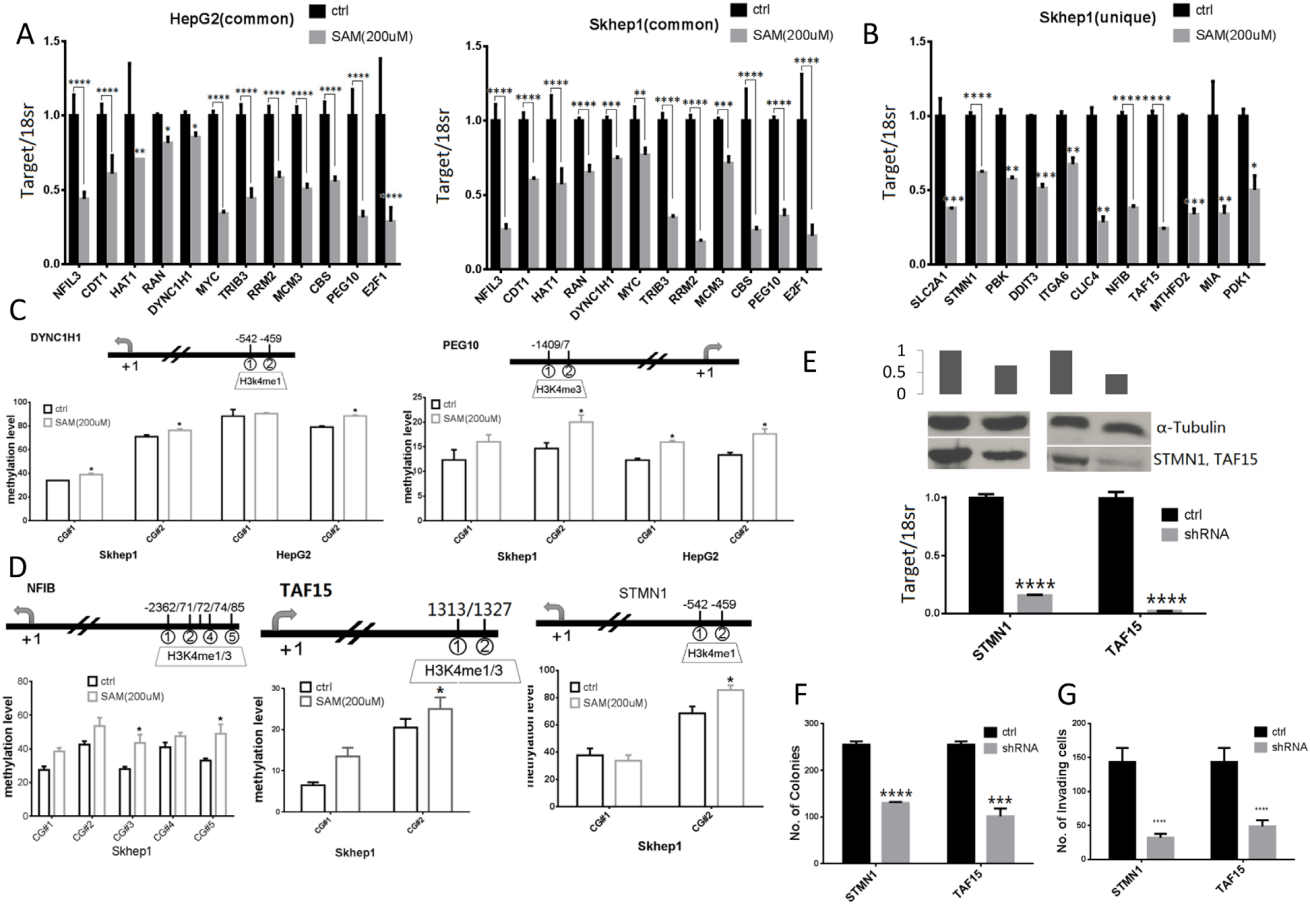
Expression, methylation and functional role of SAM target genes as determined by QPCR, pyrosequencing and shRNA depletion **(A)** Normalized mRNA expression quantified by RT-QPCR for genes whose expression is altered by SAM treatment in both HepG2 and SKhep1: *NFIL3, CDT1, HAT1, RAN, DYNC1H1, MYC, TRIB3, RRM2, MCM3, CBS, PEG10* and *E2F1*. **(B)** Normalized mRNA expression quantified by RT-QPCR for genes whose expression is altered by SAM treatment in SKhep1 cells: *SLC2A1, STMN1, PBK, DDIT3, ITGA6, CLIC4, NFIB, TAF15, MTHFD2, MIA* and *PDK1.* (Experiments were performed in triplicate and expression levels were normalized to 18S rRNA values). **(C-D)** Average methylation levels at indicated CpG sites as determined by pyrosequencing in the promoters of *DYNC1H1,* and *PEG10* (C) in both HepG2 and SKhep1 cell lines and *NFIB, TAF15, STMN1* (D) in SKhep1 (see Supplementary Figure 12 for additional methylation analysis). Positions (relative to TSS) of CpGs that were pyro sequenced are indicated above the chart. **(E)** Expression of shRNA depleted genes in SKhep1 cells was quantified by qPCR and western blot analysis after infection with *STMN1, TAF15* and scrambled shRNA lentiviral vectors. **(F-G)** anchorage independent growth was measured by soft-agar assay and invasiveness using ECM550 invasion assay kit after depletion of *STMN1* and *TAF15* as described in “Material and Methods”. All results represent mean ±SD of three determinations in either two independent experiments; ^****^, P<0.0001; ^***^, P < 0.001; ^**^, P < 0.01; ^*^, P < 0.05.

We used QRT-PCR to validate changes in expression in genes whose a. function was shortlisted by IPA analysis as related to proliferation, b. were upregulated in both SKhep1 and HepG2 cells relative to NorHep and c. were downregulated by SAM. We also validated that SAM downregulated metastasis related genes that were upregulated in untreated SKhep1 relative to NorHep (Table 1). The QRT-PCR assay confirmed that expression of all the tested genes was significantly downregulated by SAM (Figure 6A). We measured DNA methylation using pyrosequencing, focusing on differentially methylated CGs located at H3k4me1 or H3k4me3 peaks in proximity with the TSS of three genes (Figure 6C *DYNC1H1* and *PEG10*, Supplementary Figure 13 *RAN)*.

QRT PCR validated that SAM downregulated 11 metastasis related genes in SKhep1 cells (Figure 6B). We validated by pyrosequencing hypermethylation of 4 genes in response to SAM treatment (Figure 6D and Supplementary Figure 13B).

To further confirm that genes that were uniquely upregulated in SKhep1 and silenced and hypermethylated in response to SAM treatment were functionally involved in the invasive phenotype we depleted in SkeHep1 cells the mRNA of two genes selected from Table 1 and Figure 6B, 6D, *STMN1* and *TAF15* and measured the effect of depletion of these genes on the transformation and invasive phenotypes. These two genes were selected for the biological plausibility of their involvement in oncogenesis. *Stathmin1 (STMN1)* is a candidate oncogene whose activity is influenced by p53, p27, and the P13K/Akt pathway and is involved in metastasis [27–30]. *TATA-box binding protein associated factor 15* (*TAF15)* is a member of the FET family of RNA- and DNA-binding proteins which play a role in gene transcription, is a member of the TFIID transcription initiation complex and was shown to undergo translocation in acute leukemias and sarcomas [31–34]. Our results show that depletion of either *STMN1* or *TAF15* mRNA in SKhep1 cells (Figure 6E, mRNA; protein) results in inhibition of anchorage-independent colonies and cell invasiveness as measured by Boyden chamber assay (Figure 6F, 6G). These results support the hypothesis that these genes effected by SAM are potentially involved in cancer and invasion.

## DISCUSSION

SAM is biosynthesized in cells by a highly regulated process that is attentive to monocarbon metabolism and dietary supply of vitamins such as vitamin B12 and folic acid [35, 36]. SAM is a methyl donor in numerous methylation reactions including epigenetic methyl transferase reactions such as DNA methylation and histone methylation [19, 37–40]. Early studies have shown that manipulations that reduce methyl supply in the diet such as ethionine [11], choline deficient diets [12], methyl deficient diets [13] or ethanol [14], induce liver cancer in animal models, while pretreatment with SAM can protect animals from developing hepatocellular carcinoma initiated by 1,2-dimethylhydrazine (1,2-DMH) and promoted with dietary Orotic Acid [15, 16]. Studies suggested that methyl deficient and hypomethylating diets cause activation by demethylation of oncogenes [41–46]; SAM supplementation might protect from this loss of methylation. Later papers pointed to another interesting role for hypomethylation in turning on pro-metastatic genes [47] and the possibility that SAM might inhibit this hypomethylation, downregulate pro-metastatic genes and impede cancer metastasis [25, 48, 49]. However, analysis of just few genes provides anecdotal information on the impact that an agent like SAM might have on the phenotype.

Potential adverse effects of SAM should be considered as well. For example, SAM might promote methylation and downregulation of tumor suppressor genes and other critical genes that block cancer development and thus trigger cancer rather than inhibiting it, raising questions about the utility of a general compound such as SAM for preventing and treating cancer. Moreover, there is no prior reason to suggest that SAM supplementation would have no impact on normal untransformed cells and this possibility has not been critically assessed.

We therefore asked here the following questions; first, does SAM selectively inhibit growth of cancer cells and invasion of invasive liver cancer cells? Second, does SAM have a different impact on the transcriptome and methylome in normal liver primary cells, invasive cancer cells and noninvasive liver cancer cells? Third, does the cell-specific impact that SAM has on the transcriptome and methylome partly explain different phenotypic effects of SAM on cancer cells, invasive cancer cells and normal liver cells?

We show here that SAM has a strong inhibitory effect on cell growth of two different liver cancer cells, HepG2 a noninvasive HCC and SKhep1 an invasive adenocarcinoma, but has a very weak effect on the growth rate of NorHep cells (Figure 1). SAM inhibits the invasiveness of SKhep1 cells but doesn’t alter the noninvasive phenotype of primary liver cells or HePG2 cells. We reasoned that SAM has different phenotypic effects in these three liver cell types because the transcriptomic landscape that it acts upon is different. An analysis of gene expression pathways that are either upregulated or downregulated in these cell lines relative to NorHep reveals a marked activation of cell proliferation pathways in both cancer cell lines and a specific activation of the invasion pathways in SKhep1 cells. SAM acts on these pathways that differentiate cancer cell lines from normal cells and downregulates them (Figure 2). Although SAM has a broad impact on the transcriptome and effects genes unrelated to cancer, it significantly reverses pathways that are upregulated in liver cancer cell lines relative to primary liver cells. Thus, although we are using a common general agent that acts upon both normal and cancer cells, the transcriptional landscape that it acts upon is different and it might define the final transcriptomic and phenotypic output.

SAM can alter DNA methylation, possibly through altering the SAM/SAH ratio in the cell which leads to inhibition of DNA methyltransferase activity [50]. Indeed, our data show that SAM treatment causes widespread changes in DNA methylation across many chromosomes (Figure 3). Notably however, the changes in DNA methylation occur in both directions, hyper- and hypo-methylation. Thus, the genome-wide effects of SAM suggest that it is a modulator of the methylome rather than a pure “hypermethylating” agent.

Careful analysis suggests that in spite of the broad effects of SAM, it nevertheless significantly affects genes with a “cancer specific” DNA methylation profile; genes whose state of methylation is altered in cancer cell lines in comparison with primary liver cells (Figure 4). We reasoned that changes induced by SAM in this group of genes that exhibit cancer cell line specific methylation and transcription profiles is functionally related to the phenotypic changes triggered by SAM (Figure 5). Genes that are hypomethylated and upregulated in the cancer cell lines relative to primary cells and become hypermethylated and downregulated in response to SAM are enriched in the pathway of proliferation in both cancer cell lines and in the invasion pathway in SKhep1 cells, consistent with the phenotypic effects of SAM (Figure 5). Thus, the alterations in DNA methylation profiles caused by SAM, similar to the changes in transcription, reflect the cell-specific DNA methylation landscape that the general methylation agent SAM is acting upon. It should be noted however that our study is limited by the fact that it examined two liver cancer cell lines and a primary liver cell culture. Future studies should examine whether the conclusions derived from the cell lines examined here hold for a wide range of liver cancer cells.

We demonstrate that SAM-targets that are upregulated in cancer cells and methylated and downregulated by SAM play a causal role in cancer proliferation and invasiveness (Figure 6). Knockdown of two such genes *STMN1* and *TAF15* inhibits both invasiveness and the proliferative capacity of the cells (Figure 6). The workflow that we used here could be utilized as a general pipeline towards identification of genes that are potential targets for anti-metastasis and anticancer agents.

SAM surprisingly causes both increase and decrease in DNA methylation as well as an increase and a decrease in gene expression, which is counterintuitive since SAM is a methyl donor that should stimulate rather than suppress DNA methylation. The mechanisms that might be responsible for this are unclear and at this stage we can only speculate. Demethylation might be an indirect consequence of changes in gene expression caused by SAM or could be downstream to enhanced methylation of H3 histones K4 residues which can result in activation of enhancers or promoters. Future studies examining the impact of SAM treatment on histone methylation might be required to address this question.

Although we were able to show selectivity of SAM effects on proliferation and invasiveness to cancer cell lines, our study reveals changes in the transcriptome in normal untransformed cells as well (Figure 2). We were not able to evaluate the effects of SAM on phenotypes other than proliferation or invasion in this study but they should be addressed in animal studies to rule out other potential adverse effects of SAM therapy. Our study points to the possibility that transcriptome profiles are altered by SAM that might have effects on normal tissue function, which should be examined in future detailed dose response studies in whole animals and clinical trials.

In summary, SAM is an attractive anticancer compound since it is a nutritional supplement with limited documented toxicities. Although SAM is a general methyl donor, differences in the transcription and methylation landscape in cancer and untransformed cells and the different way by which they respond to SAM intervention results in gene expression outcomes that are specific to cancer and metastasis and are associated with selective phenotypic outcomes. Our results might be applicable to other general epigenetic modifiers which might nevertheless cause specific outcomes in cancer cells that are predetermined by the epigenetic matrix that they are acting upon.

## MATERIALS AND METHODS

### Cell culture and treatments

Human HCC cell lines HepG2, adenocarcinoma SKhep1cells were purchased from ATCC (HB8065, HTB52, respectively), human untransformed primary hepatocytes (normal hepatocytes, NorHep) were obtained from Celprogen (33003-02). S-adenosylmethionine chloride (SAM) (Life Science Laboratories, Lakewood, NJ)was prepared in buffer containing 0.005M sulphuric acid and 10% ethanol. 200μm SAM or equivalent volume of buffer (control) were added to regular culture medium. Media were refreshed daily over a period of 5 days.

### Viability, invasion, and anchorage-independent growth assays

Cell viability was determined by the Trypan blue (Sigma-Aldrich) exclusion assay. Cells were harvested after 5 days of treatment. Cell invasiveness was evaluated by the Boyden Chamber Cell Invasion Assay Kit ECM550 (Chemicon Int.). Briefly, 50,000 cells resuspended in serum-free media were added to the inserts which were dipped in the lower chamber containing complete media. Following 24h incubation at 37°C, invasive cells were stained and counted under the microscope. Additionally, 50,000 viable cells (as determined by Trypan blue) resuspended in complete media were added to a six-well plate (in absence of the insert) and were counted concurrently following 24h incubation period to determine effects on cell viability.

Anchorage-independent growth on soft agar, a measure of transformation *in vitro* was determined as described [51]. 3,000 viable cells treated for 5 days with SAM were seeded into soft agar. The total number of colonies (>10 cells/colony) was counted under the microscope after three weeks of plating.

### DNA/RNA extraction, quantitative real-time PCR and western blot

DNA and RNA was extracted using AllPrep DNA/RNA/miRNA Universal Kit (Qiagen) according to the manufacturer’s protocol. Quantitative real-time PCR (QPCR) reaction was carried out in Light Cycler 480 machine (Roche) using forward and reverse primers listed in Supplementary Table 1 and quantified using Roche LightCycler 480 software second derivative method.

Western blot analysis was performed as described [52] using 50-100 μg of protein samples fractionated on a12% SDS-polyacrylamide gel (SDS-PAGE). The proteins were immunoblotted with anti-STMN1 (ab131481, ABcam) or anti-TAF15 (MABE450, Millipore) antibody at 1:500 dilution, followed by a secondary anti-rabbit (Cat#A0545) or anti-mouse (Amersham Biosciences) IgG antibody at 1:5000 dilution. The membranes were hybridized with an anti-α-Tubulin antibody as a loading control (Cat#T9026, Sigma-Aldrich).

### Bisulfite conversion, pyrosequencing

DNA samples (1μg) were subjected to bisulfite conversion using the EZ DNA Methylation-Gold Kit (Zymo Research, D5005) according to the manufacturer’s protocol. Bisulfite converted target sequences were amplified with HotStar Taq DNA polymerase (Qiagen) using biotinylated primers listed in Supplementary Table 2. Pyrosequencing was performed in PyroMarkTMQ24 (Biotage) and data was analyzed using the PyroMarkTMQ24 software (Colella, Shen et al. 2003).

### shRNA inhibition

For STMN1 and TAF15 depletion we used lentivirus-mediated human pGIPZ shRNA plasmids and control pGIPZ-scrambled shRNA (Open Biosystems) (Supplementary Table 2). shRNAs were selected based on knockdown efficiency in the SKhep1 cells; *STMN1* was targeted with ShSTMN1#V3LHS_383505 and *TAF15* was targeted with ShTAF15#V2LHS_172493 (Supplementary Table 1 for sequences).

### RNA sequencing and data analysis

RNA (4 μg) was prepared for sequencing using the TruSeq RNA Sample Prep Kit (Illumina, San Diego, USA) following the manufacturer’s protocol and sequenced in duplicate using Illumina HiSEQ2K platform (Illumina, San Diego, USA)(50bp pair-end reads). Around 30 million reads were obtained per sample (Supplementary Table 3).

Fastqc was used to quality control (QC) RNAseq data, paired end reads were aligned to the human reference sequence (hg19, Feb. 2009) with TopHat 2.0.9 [53] with default setting. Aligned bam files were assessed using cufflinks v.2.2.1 [54] to estimate expression levels (FPKM). Differentially expressed genes (DEGs) were delineated by applying the threshold false discovery rate (FDR) <0.05. Reads counts were obtained using HTSeq-count (1.0)[55]. Extreme low expressed genes < 2 count-per-million (CPM) were filtered using EdgeR package [56].

### DNA capture bisulfite sequencing

Target DNA fragments were captured using the human SeqCap Epi CpGiant Enrichment Kit (Nimblegen, USA) interrogating 5.5 million CpG sites covering promoters and regulatory sequences in human genome according to the manufacturer’s recommendations. Sequencing was performed on the Illumina HiSEQ2K platform using a standard 50 cycle paired-end read sequencing protocol according to the manufacturer’s recommendations.

### Bisulfite sequencing data analysis

The raw data was processed as recommended by Sequencing Solutions Technical Note from Roche for SeqCap Epi CpGiant bisulfite sequencing data analysis (https://sftp.rch.cm//diagnostics/sequencing/literature/nimblegen/07292163001_NG_SeqCap_TchNote_EvalEpiData.pdf). Methylation difference was calculated using methylKit [57] according to the users guide (coverage count >5). The differentially methylated CpGs were extracted with a q-value <0.05 and delta methylation >15%. The differentially methylated CpG sites (DMC) was annotated with CHIPseeker package [58].

### Statistical analysis

Statistical analysis of qPCR and pyrosequencing data was performed using an unpaired t test with two tailed distributions. The results were considered statistically significant when *P* <0.05.

Ingenuity Pathway Analysis (IPA) program (http://www.ingenuity.com/index.html) was used to compute enriched gene networks, functional categories, canonical pathways and upstream regulators. Heatmaps were created using GeneE (http://www.broadinstitute.org/cancer/software/GENE-E/doc.html). GSEA was used to examine gene-set enrichment of differentially methylated genes in response to SAM and their expression profile [59].

## SUPPLEMENTARY MATERIALS FIGURES AND TABLES














