# Supplementary material for: S-adenosyl-methionine (SAM) alters the transcriptome and methylome and specifically blocks growth and invasiveness of liver cancer cells
Source: Oncotarget. 2017 Dec 5;8(67):111866–81. doi: 10.18632/oncotarget.22942 (PMC5762365; doi:10.18632/oncotarget.22942)
Supplement: Supplementary file 1 [file oncotarget-08-111866-s001.pdf]

S-adenosyl-methionine (SAM) alters the transcriptome and methylome and specifically blocks growth and invasiveness of liver cancer cells

SUPPLEMENTARY MATERIALS

Cell culture and drug treatments

HepG2 and SkHep1 cells were maintained in MEM medium (Gibco), supplemented with 2 mmol/L glutamine (Sigma-Aldrich), 10% fetal bovine serum (FBS; Gibco), 1 U/mL penicillin, and 1 µg/mL streptomycin (Gibco). NorHep cells were maintained in human hepatocyte cell culture complete medium (Celprogen). Cells were grown in a humidified atmosphere of 5% carbon dioxide at 37°C. Following 3 to 5 minute incubation with Trypan blue after trypsinization, the viable cells were counted under the microscope.

The ability of cells treated with SAM to invade through extra cellular matrix was evaluated by the Cell Invasion Assay Kit (Chemicon Int.). The kit utilizes a reconstituted basement membrane matrix of proteins derived from Engelbreth–Holm–Swarm (EHS) mouse tumor. Briefly, 50,000 cells resuspended in serum-free media were added to the inserts dipped in the lower chamber containing complete media. Following 24h incubation at 37°C, invasive cells were stained and counted under the microscope. Additionally, 50,000 viable cells (as determined by Trypan blue) resuspended in complete media were added to a six-well plate and were counted following 24h incubation period concurrently with measuring invasiveness to measure cell viability.

To determine anchorage-independent growth on soft agar, a measure of transformation *in vitro* [1], 3,000 viable cells treated for 5 days with SAM were seeded into soft agar and plated in triplicate in a 6-well dishes containing 4mL of complete medium with 0.33% BD Bacto™ agar solution at 37°C as previously described [2]. The total number of colonies (>10 cells/colony) that formed on soft agar was counted under the microscope after three weeks of plating.

DNA/RNA extraction, quantitative real-time PCR and western blot

DNA and RNA was extracted using AllPrep DNA/RNA/miRNA Universal Kit (Qiagen) according to the manufacturer's protocol. One microgram of total RNA served as a template for cDNA synthesis using 20U of AMV reverse transcriptase (Roche Diagnostics), as recommended by the manufacturer. The quantitative real-time PCR (QPCR) reaction was carried out in Light Cycler 480 machine (Roche) using forward and reverse primers listed in Supplementary Table 1. Quantification was performed using Roche LightCycler 480 software second derivative method.

Western blot analysis was performed as described [3]using 50-100 µg of protein and a 12% SDS-polyacrylamide gel electrophoresis (SDS-PAGE). The proteins were immunoblotted with anti-STMN1 (ab131481, ABcam) or anti-TAF15 (MABE450, Millipore) antibody at 1:500 dilution, following by a secondary anti-rabbit (Cat#A0545) or anti-mouse (Amersham Biosciences) IgG antibody at 1:5000 dilution. The membranes were blotted with an anti-α-Tubulin antibody as a loading control (Cat#T9026, Sigma-Aldrich).

shRNA inhibition

For *STMN1* and *TAF15* depletion we used the lentivirus human pGIPZ shRNA plasmids and control pGIPZ-scrambled shRNA (Open Biosystems) (Supplementasy Table S2). Lentiviruses were assembled using the following three vectors: GFP expression pGIPZ transfer vector—includes the insert (Open Biosystems); pMD2.G (VSV-G envelope expressing plasmid); PAX (packaging plasmid). The day before transfection, 10^6 HEK293T cells were plated in a 10 cm dish (20–30% confluence). Next day, 5 µg of each vector were transfected using FuGene HD transfection reagent (Roche) according to the manufacturer's protocol. Cells were incubated for 48 h followed by collection of the medium containing the virus, filtered and used to infect the target cells. Selection with 1 mg/ml puromycin (Sigma) was started 48h post infection. Specific shRNAs were selected based on knockdown efficiency in the specific cell line; *STMN1* was targeted with ShSTMN1#V3LHS_383505 and *TAF15* was targeted with ShTAF15#V2LHS_172493 (Supplementary Table 1 for sequences).

RNA sequencing and data analysis

RNA (4 µg) from SAM and SAM buffer (control) treated cells was processed using the TruSeq RNA Sample Prep Kit (Illumina, San Diego, USA) following

the manufacturer’s protocol. Briefly, polyA mRNA was purified and fragmented. Then first and second strand cDNA synthesis was performed, ends were repaired and adenylated, adapters were ligated, and fragments were enriched with PCR amplification. The library was validated with an Agilent Technologies 2100 Bioanalyzer (Agilent Technologies, Palo Alto, CA). RNA-seq preparations were sequenced using Illumina HiSEQ2K platform (Illumina, San Diego, USA). A total of 50bp pair-end reads were generated. Duplicate samples were sequenced. Around 30 million reads were obtained per sample (Supplementary Table 3).

Fastqc was used to QC RNAseq data. Raw reads were trimmed sequentially for adapter contamination. The paired end reads were aligned to the human reference sequence (hg19, Feb. 2009) with TopHat 2.0.9 [4] with default setting, except for the option -g. Aligned bam files were assessed using cufflinks v.2.2.1 [4]to generate a transcriptome assembly and to estimate the expression level (FPKM) of all detected isoforms. FPKM was calculated as number of end-paired reads (a single fragment per end-paired reads) mapped to a gene divided by the number of all fragments mapped to the genome (in million) and the length of RNA (in KB). The tagwise dispersion were estimated and then used for logFC (log_2 fold change) estimating and testing. Differentially expressed genes (DEGs) were extracted by applying the threshold false discovery rate (FDR) of less than 0.05 to adjusted *P* values using Fisher’s exact test. Furthermore, Reads counts were obtained from the mapping results by using HTSeq-count (1.0) [5]. Extreme low expressed genes < 2 count-per-million (CPM) were filtered using EdgeR package [6].

DNA capture bisulfite sequencing

DNA (5µg) from SAM and SAM buffer (control) treated cells was used for preparation of DNA capture bisulfite sequencing library. The DNA libraries were prepared using Illumina's paired-end sequencing DNA sample preparation kit according to the manufacturer's protocol. Target DNA fragments were captured using the human SeqCap Epi CpGiant Enrichment Kit (Nimbergen, USA) according to the manufacturer's recommendations. SeqCap Epi CpGiant (Nimblegen, Roche) interrogates more than 5.5 million CpG sites covering promoters and regulatory sequences in human genome. In order to capture bisulfite converted DNA, probes are designed to hybridize to both strands of fully methylated, partially methylated and fully unmethylated derivatives of the genomic target and then pooled together. DNA libraries were quality-checked and quantified on Agilent 2100 Bioanalyzer. Sequencing was performed on the Illumina HiSEQ2K platform using a standard 50 cycle paired-end read sequencing protocol and Illumina's sequencing reagents according to the manufacturer's recommendations.

Bisulfite sequencing data analysis

The raw data was processed as recommended by Sequencing Solutions Technical Note from Roche for SeqCap Epi CpGiant bisulfite sequencing data analysis (https://sftp.rch.cm//diagnostics/sequencing/literature/nimblegen/07292163001_NG_SeqCap_TchNote_EvalEpiData.pdf). Briefly, the FASTQ reads were trimmed using Trimmomatic0.30 [7] and aligned with BSMAP2.74 [8] to the human reference sequence (hg19, Feb. 2009). After removal of duplicated reads and filtering for properly paired reads from BSMAP mapping results, methylation ratios were extracted using python script methratio.py from the BSMAP package. Methylation difference was calculated using methylKit [9] according to user guide (coverage count >5). The differentially methylated CpGs were extracted with a q-value <0.05 and delta methylation >15%. The differentially methylated sites were annotated with CHIPseeker package [10].

Statistical analysis

Statistical analysis of pyrosequencing, QPCR, invasion, soft agar and cell viability assays was performed using an unpaired t test with two tailed distribution. The results were considered statistically significant when *P* <0.05.

The Ingenuity Pathway Analysis (IPA) program (http://www.ingenuity.com/index.html) was used to compute enriched gene networks, functional categories, canonical pathways and upstream regulators. Heatmaps were created using GeneE (http://www.broadinstitute.org/cancer/software/GENE-E/doc.html).

Gene set enrichment analysis of expression

GSEA (GSEAv2.2.3) [11] was used to examine the gene sets that are enriched with genes whose expression increases after SAM treatment that are significantly downregulated in cancer vs. normal cells and genes whose expression decreases after SAM treatment that are significantly upregulated in cancer vs. normal cells. GSEA was also used to examine the gene expression profiles of gene sets enriched with genes that are either hypomethylated or hypermethylated in response to SAM. For differential expression FDR of < 0.05 and count-per-million (CPM) of >=2 were used as thresholds. For differential methylation the thresholds were q-value <0.05 and delta methylation >15% in promoter regions (-1000bp-1000bp). Significantly enriched gene sets after 1,000 permutations at FDR of <0.25 were reported.

SUPPLEMENTARY REFERENCES

1. De Larco JE, Todaro GJ. Growth factors from murine sarcoma virus-transformed cells. Proc Natl Acad Sci U S A. 1978; 75:4001-4005.
2. Slack A, Cervoni N, Pinard M, Szyf M. Feedback regulation of DNA methyltransferase gene expression by methylation. Eur J Biochem. 1999; 264:191-199.
3. Stefanska B, Huang J, Bhattacharyya B, Suderman M, Hallett M, Han ZG, Szyf M. Definition of the landscape of promoter DNA hypomethylation in liver cancer. Cancer Res. 2011; 71:5891-5903.
4. Trapnell C, Roberts A, Goff L, Pertea G, Kim D, Kelley DR, Pimentel H, Salzberg SL, Rinn JL, Pachter L. Differential gene and transcript expression analysis of RNA-seq experiments with TopHat and Cufflinks. Nat Protoc. 2012; 7:562-578.
5. Anders S, Pyl PT, Huber W. HTSeq--a Python framework to work with high-throughput sequencing data. Bioinformatics. 2015; 31:166-169.
6. Robinson MD, Mccarthy DJ, Smyth GK. edgeR: a Bioconductor package for differential expression analysis of digital gene expression data. Bioinformatics. 2010; 26:139-140.
7. Bolger AM, Lohse M, Usadel B. Trimmomatic: a flexible trimmer for Illumina sequence data. Bioinformatics. 2014; 30:2114-2120.
8. Xi Y, Li W. BSMAP: whole genome bisulfite sequence MAPping program. BMC Bioinformatics. 2009; 10:232.
9. Akalin A, Kormaksson M, Li S, Garrett-Bakelman FE, Figueroa ME, Melnick A, Mason CE. methylKit: a comprehensive R package for the analysis of genome-wide DNA methylation profiles. Genome Biol. 2012; 13:R87.
10. Yu G, Wang LG, He QY. ChIPseeker: an R/Bioconductor package for ChIP peak annotation, comparison and visualization. Bioinformatics. 2015; 31:2382-2383.
11. Subramanian A, Tamayo P, Mootha VK, Mukherjee S, Ebert BL, Gillette MA, Paulovich A, Pomeroy SL, Golub TR, Lander ES, Mesirov JP. Gene set enrichment analysis: a knowledge-based approach for interpreting genome-wide expression profiles. Proc Natl Acad Sci U S A. 2005; 102:15545-15550.

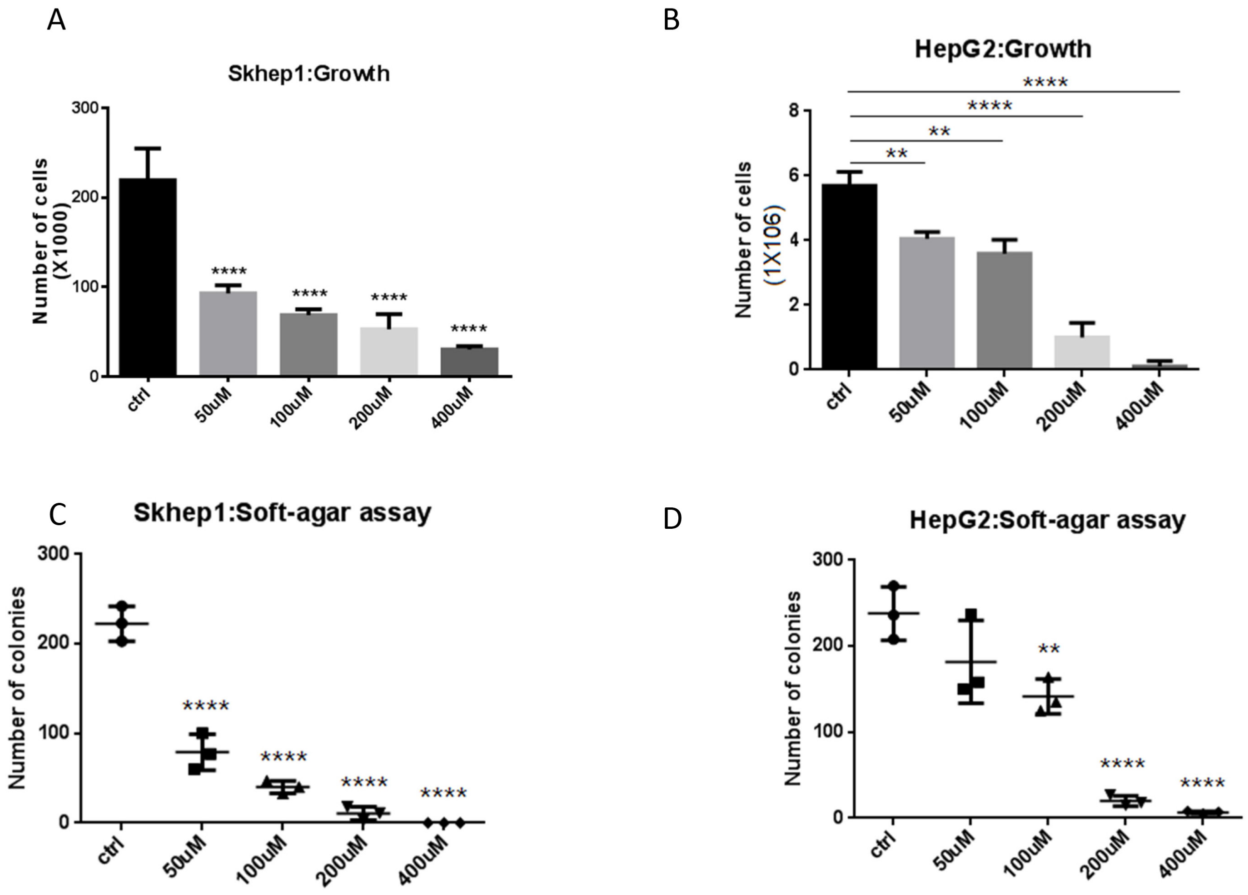

Supplementary Figure 1: Dose response of the effect of SAM on liver cancer cell viability and anchorage independent growth. Non-invasive HCC HepG2 and invasive adenocarcinoma Skhep1 cell lines were plated onto 10cm plates and treated with different concentrations (50, 100, 200, 400μM) of SAM or SAM control buffer for 5 consecutive days. Cell viability was determined by Trypan blue staining after trypsinization. Anchorage independent growth of SAM treated cells was determined using soft-agar assay by plating same numbers of viable cells onto soft agar. **(A-B)** Cell viability in presence of different doses of SAM was measured in HepG2 and Skhep1 cells respectively. **(C-D)** The effect of increasing doses of SAM on anchorage independent growth of HepG2 and SkHep1 cells. All results represent the mean ±SD of 3 determinations in either 2 or 3 independent experiments; ****, P<0.0001; ***, P < 0.001; **, P < 0.01; *, P < 0.05.

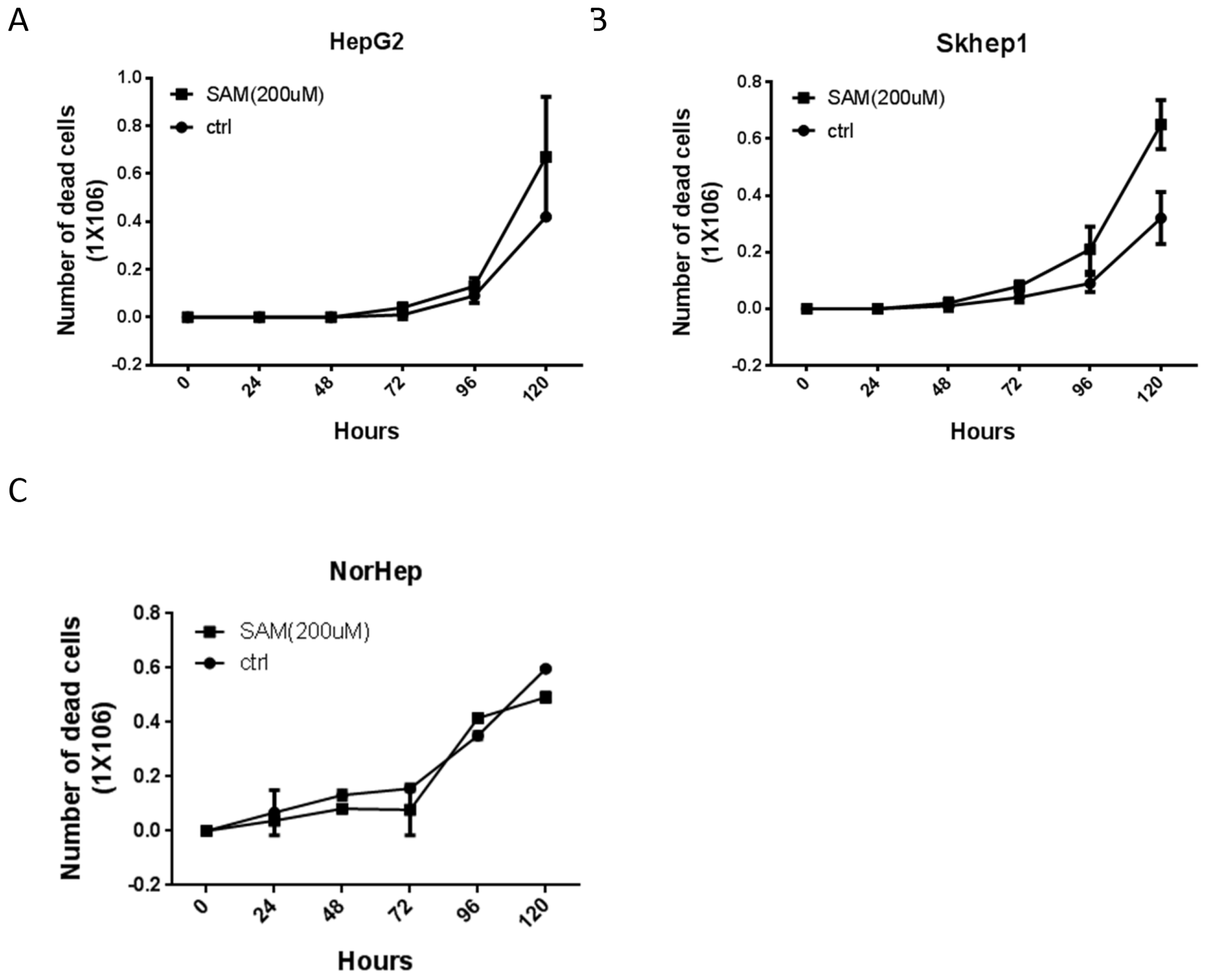

Supplementary Figure 2: SAM does not affect cell viability. HepG2 **(A)**, Skhep1 **(B)** and NorHep **(C)** cells were plated in 10cm plates and treated with 200µM of SAM or SAM control buffer. The number of dead cells was counted by trypan blue staining and Coulter counter counting. All results represent mean ±SD of 3 determinations in either 2 or 3 independent experiments.

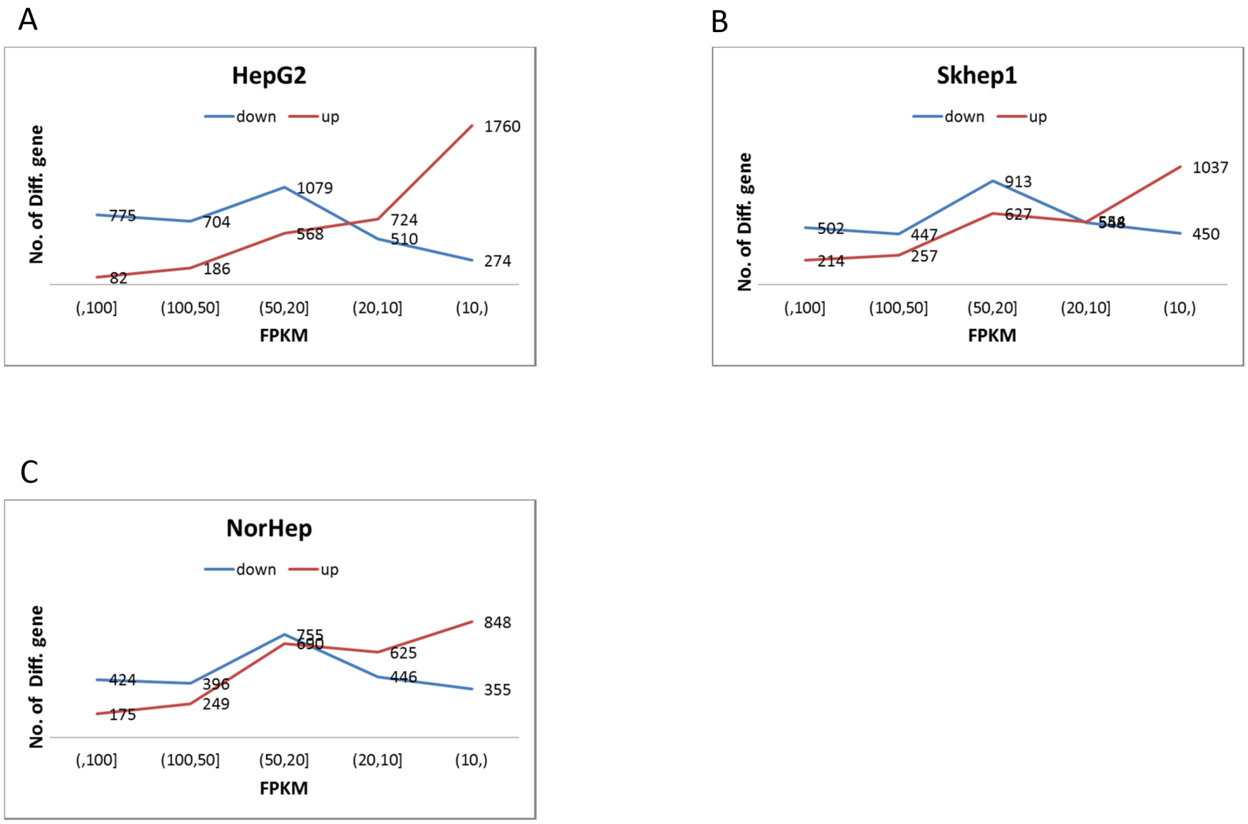

Supplementary Figure 3: Distribution of expression levels (in untreated cells) of genes that were either upregulated or downregulated by SAM treatment in HepG2 **(A)**, Skhep1 **(B)** and NorHep **(C)**. Genes that were either up- or down-regulated with SAM were separated into 5 groups according to the level of expression of the genes (FPKM) in untreated cells.

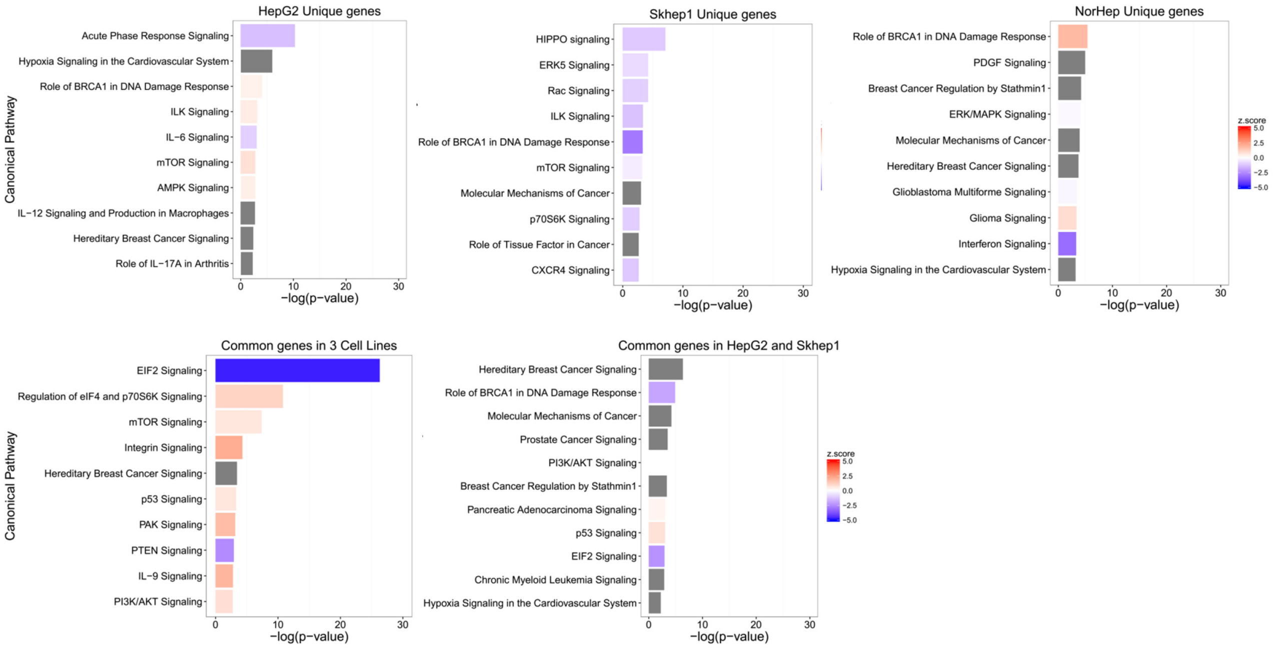

Supplementary Figure 4: The top ten Ingenuity Pathway Analysis (IPA) 'Canonical Pathways' related to cancer and enriched with differentially expressed genes (DEG) in response to SAM. uniquely in either the normal or one of the cancer liver cell lines as well as DEGs that are commonly altered in response to SAM in either the two cancer cell lines (HepG2 and SKhep1) or all three liver cell lines. Grey color no clear pattern of change in activity of pathway; Red: activation of pathway; blue: inhibition of pathway.

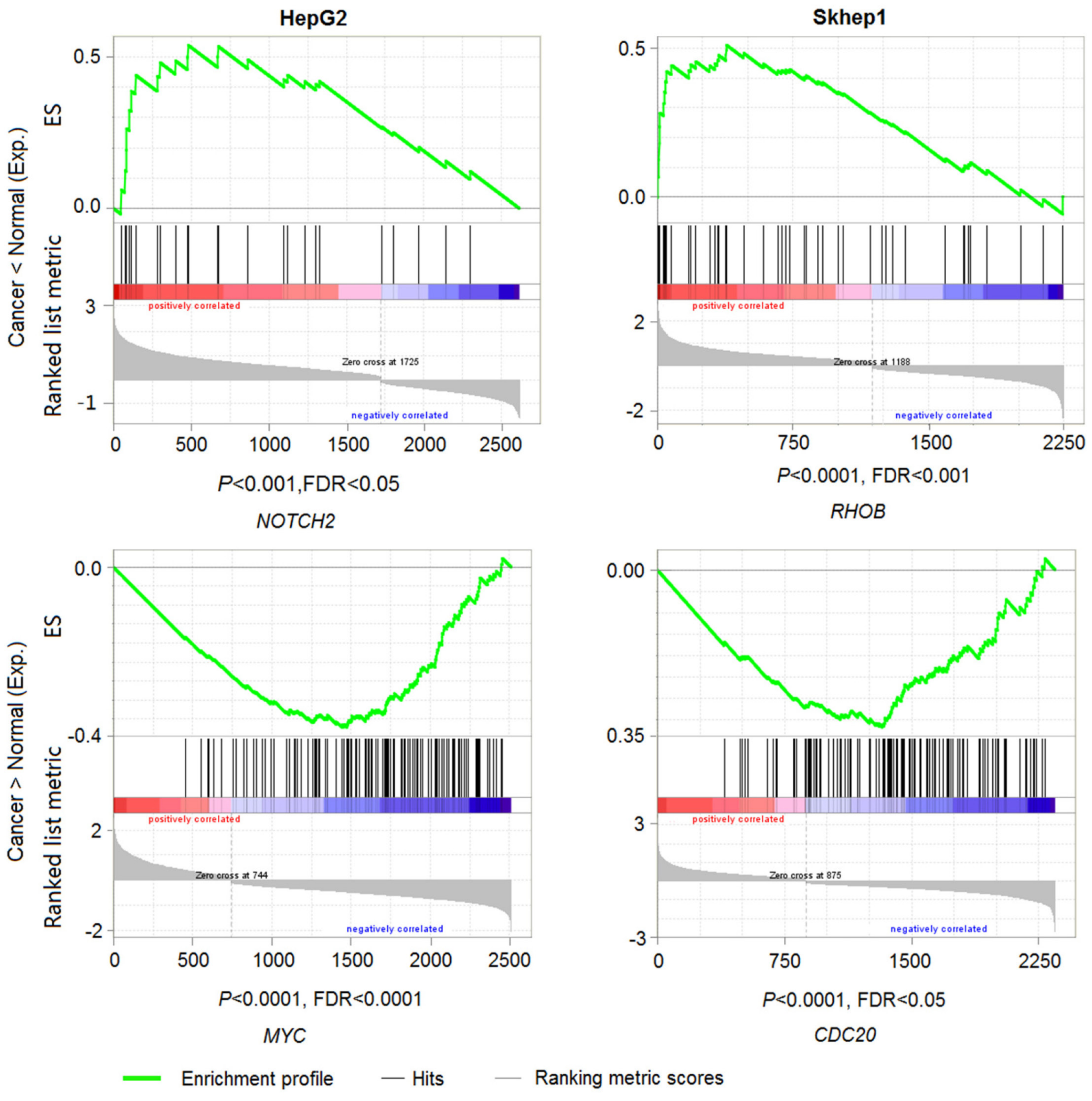

Supplementary Figure 5: GSEA analysis of the effect of SAM on genes that are differentially expressed between cancer and normal liver cell lines. Shown are enrichment score plots for the gene sets (examples gene sets) that showed significant enrichment in genes differentially methylated in response to SAM. Bars indicate genes, red-upregulated by SAM treatment, blue-downregulated by SAM. Genes whose expression is lower in untreated cancer cell lines versus normal cells were mostly upregulated by SAM (upper panel) and genes whose expression is higher in untreated cancer cell lines than normal cells were mostly downregulated (lower panel).

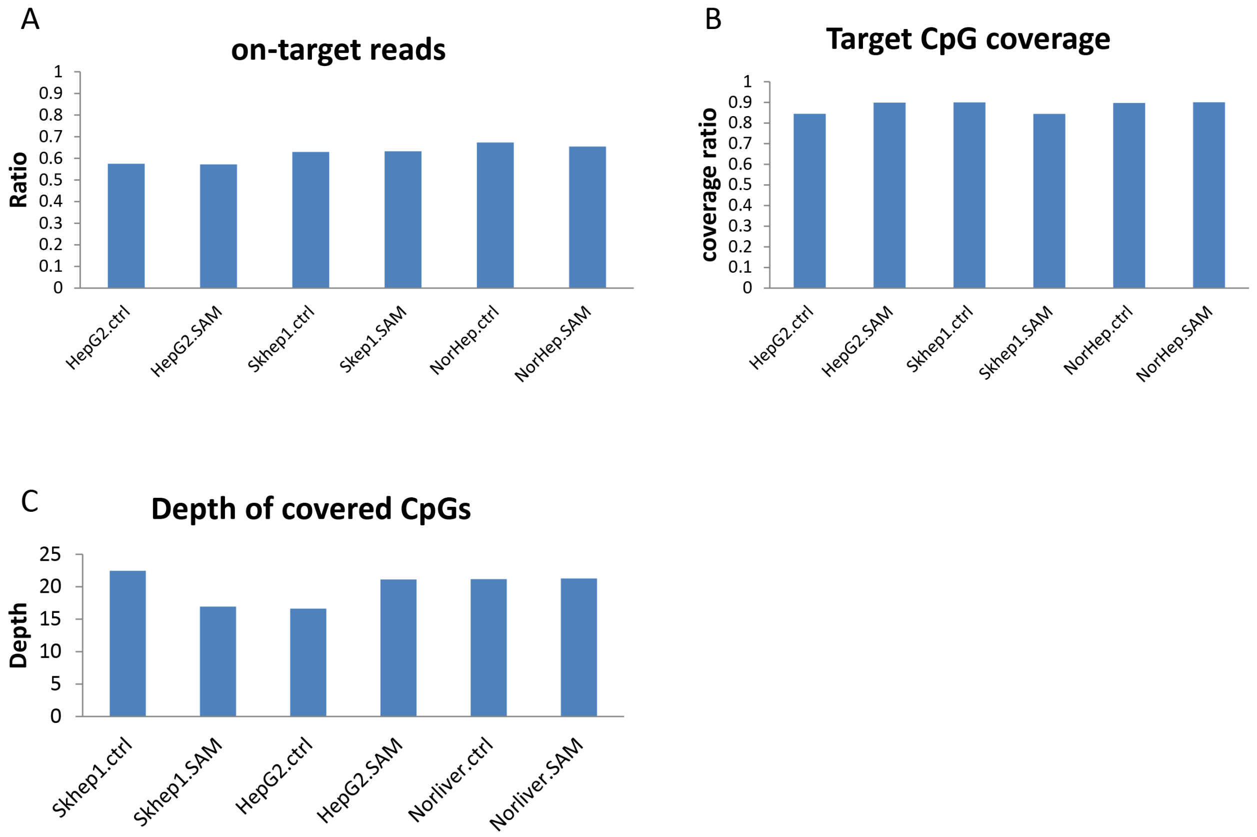

Supplementary Figure 6: Statistics of capture sequencing data. On-target reads **(A)**, coverage of on-target CpGs **(B)** and depth of the mapped CpGs **(C)**. The reads were intersected to target regions by bedtools.

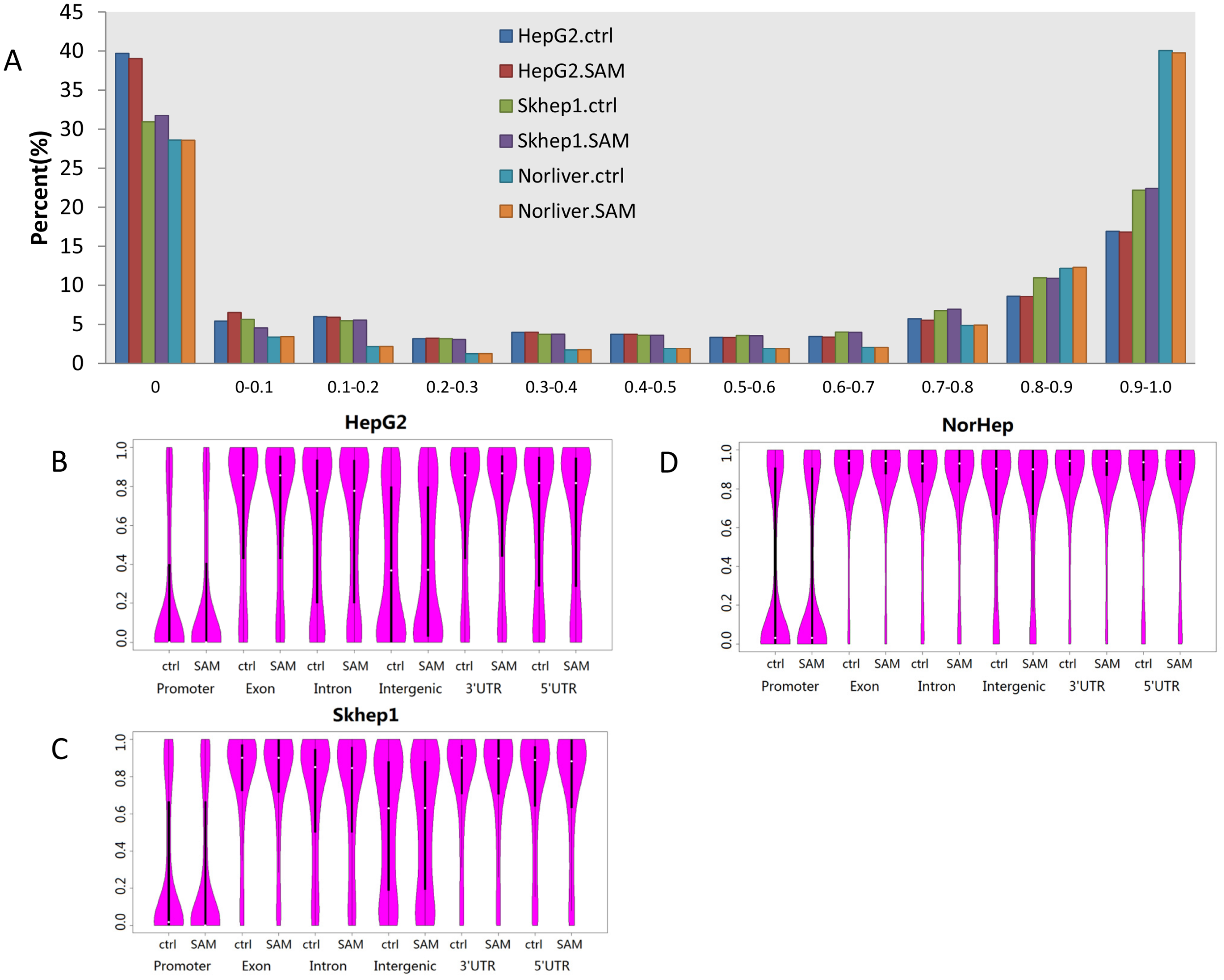

Supplementary Figure 7: Distribution of methylation levels of all the interrogated CpGs in the different cell types and across genomic features for all groups. **(A)** Histogram depicting distribution of CGs at different levels of methylation. **(B-D)** Vioplot of the distribution of methylation levels in different genomic features in control and SAM treated samples.

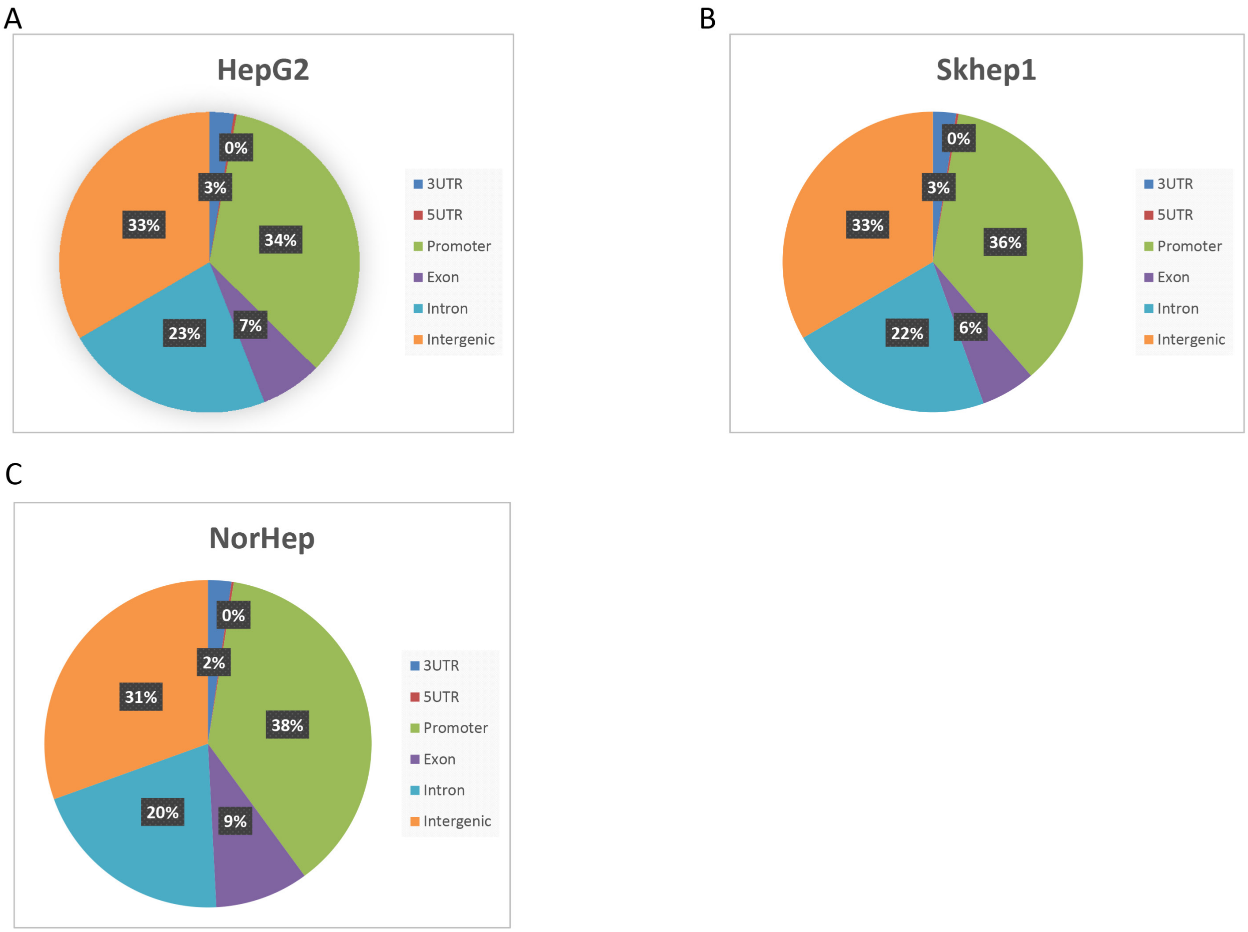

Supplementary Figure 8: Pie chart depicting distribution of differentially methylated CGs (DMC) in response to SAM across genomic features. HepG2 **(A)**, Skhep1 **(B)** and NorHep **(C)**. DMCs were annotated using the CHIPseeker package.

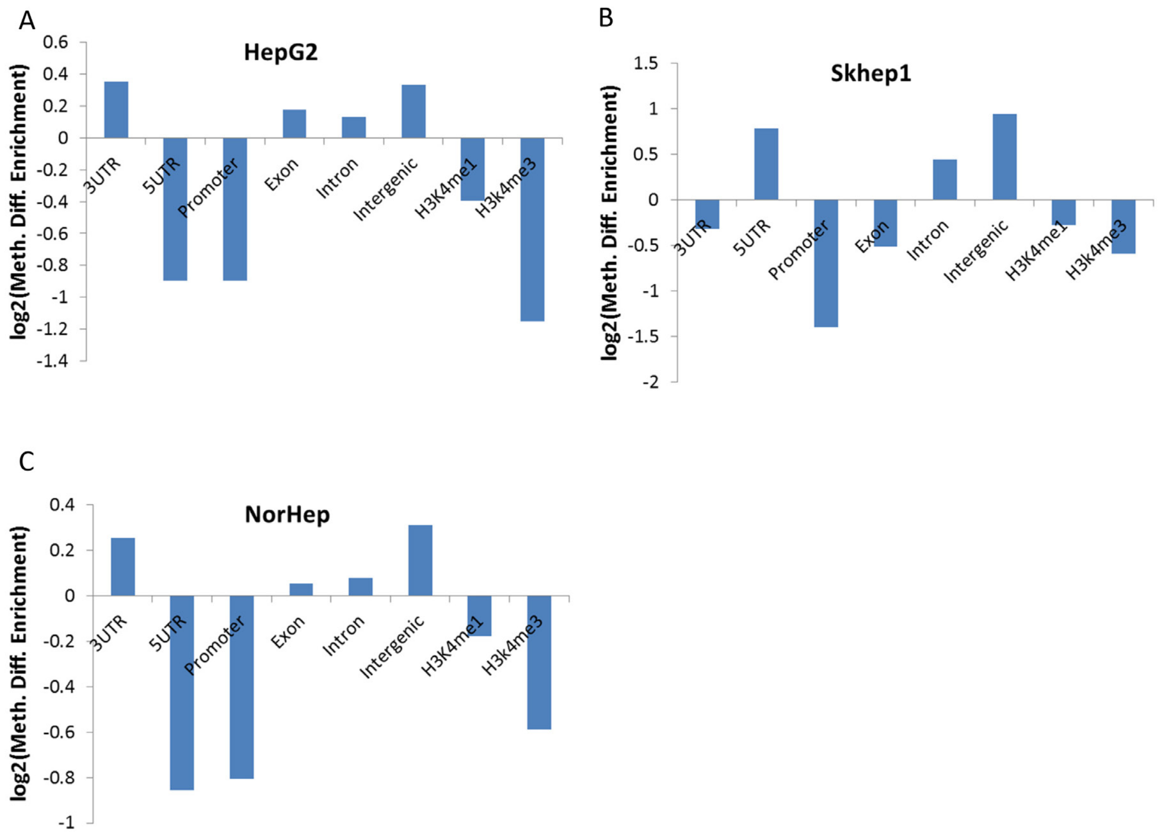

Supplementary Figure 9: Histogram depicting enrichment of DMCs (SAM/untreated) in SAM treated cells at different genomic features in HepG2 **(A)**, Skhep1 **(B)** and NorHep **(C)**. The enrichment was calculated as the number of DMCs (SAM/untreated) per genomic feature divided by the total number of interrogated CpGs in the genomic feature.

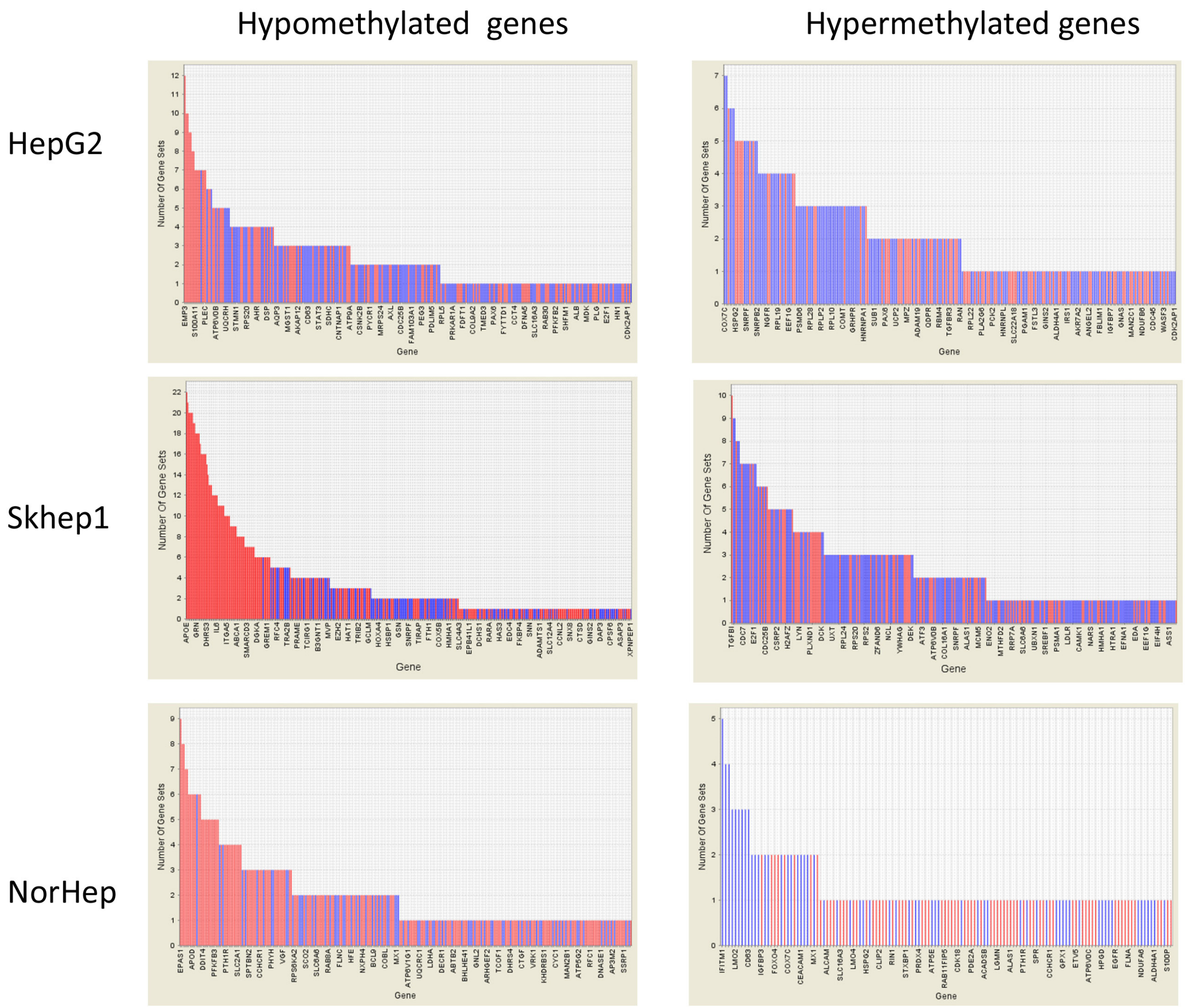

Supplementary Figure 10: GSEA analysis of genes whose state of methylation was altered by SAM. X axis: significant gene set names, Y-axis; number of genes per gene set, Red; upregulated genes, Blue: downregulated genes.

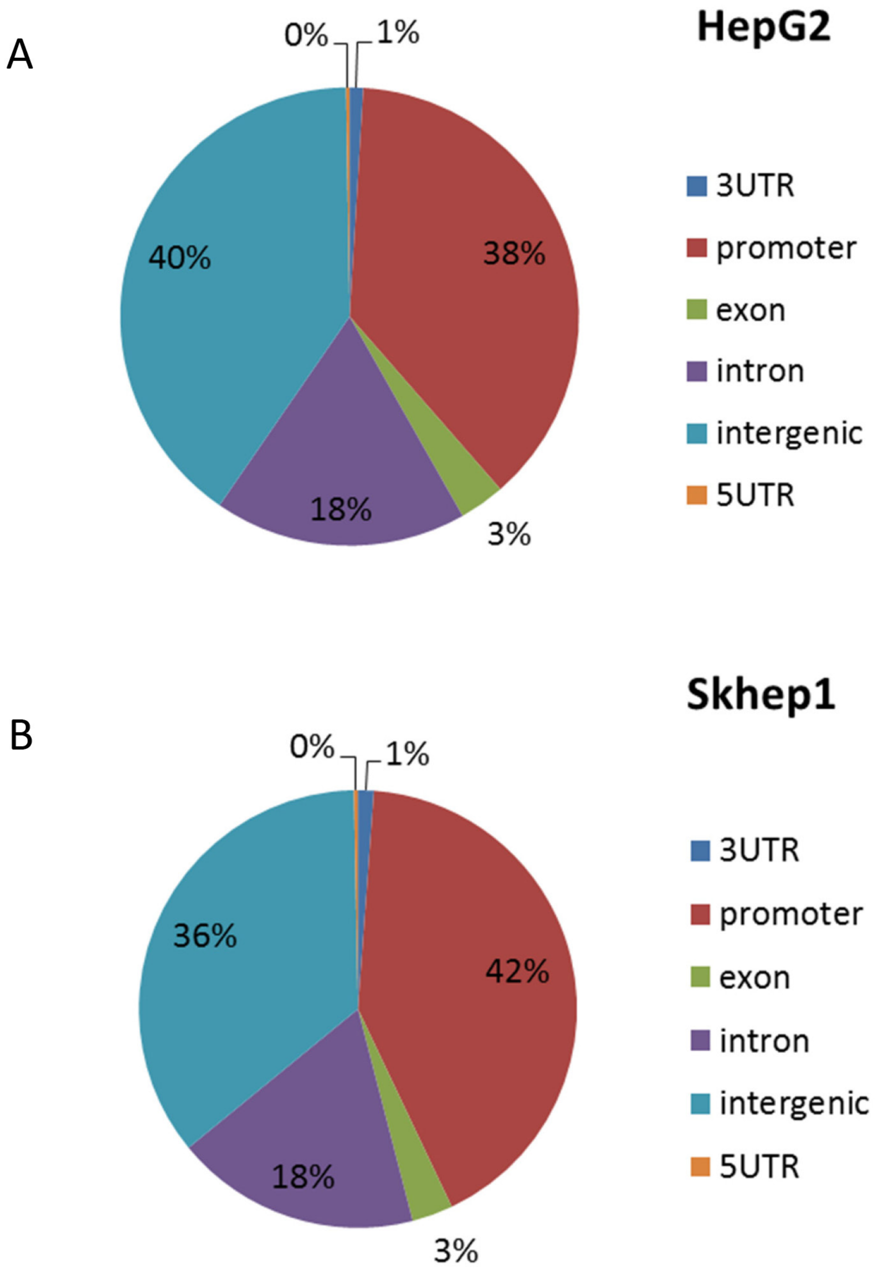

Supplementary Figure 11: Pie chart depicting distribution of differentially methylated CGs (DMC) between NorHep and cancer cell lines across genomic features. HepG2 **(A)**, Skhep1 **(B)**. DMCs were annotated using CHIPseeker package.

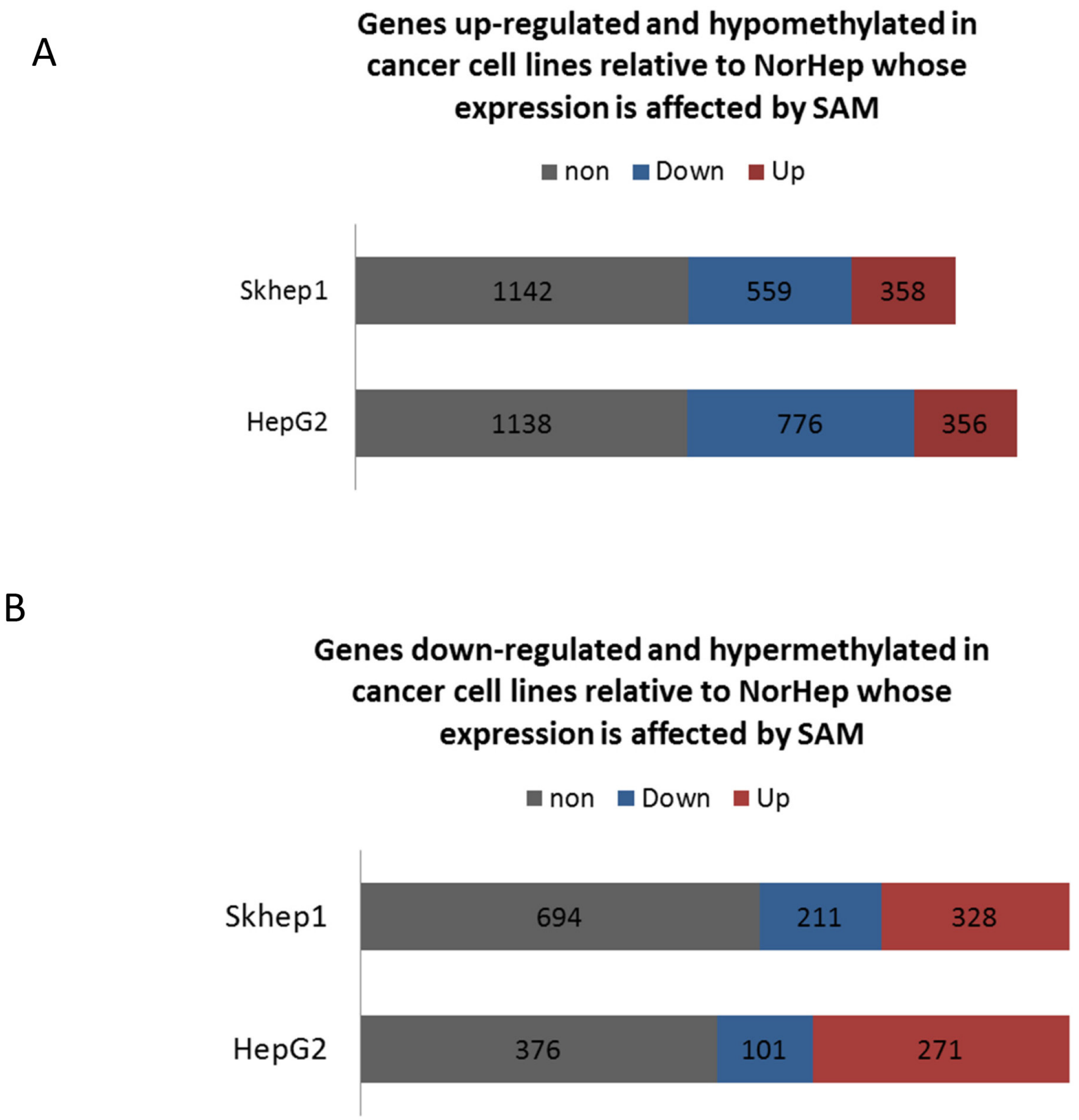

Supplementary Figure 12: Effects of SAM treatment on genes that are differentially regulated in liver cancer cell lines versus NorHep. **(A)** Genes that are hypomethylated and upregulated in cancer cell lines. **(B)** Genes that are hypermethylated and downregulated in cancer cell lines.

A

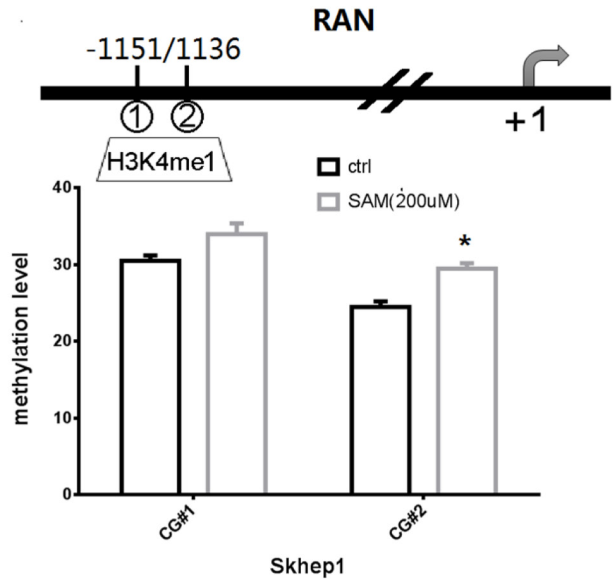

B

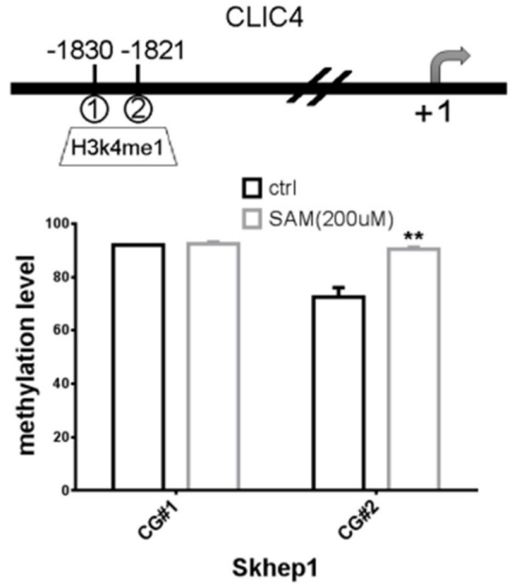

Supplementary Figure 13: Quantification of methylation by pyrosequencing of the *RAN* **(A)** and *CLIC4* **(B)** promoters. Genomic positions of CpGs that were sequenced are indicated above the chart. All results represent means ±SD of three determinations in either two or three independent experiments; ****, P<0.0001; ***, P < 0.001; **, P < 0.01; *, P < 0.05.

Supplementary Table 1: Primer sequences used for qPCR

Primers used for QPCR		
**Gene**	**Gene_id**	**Primer sequences for QPCR(5'-3')**
NFIL3	ENSG00000165030	Fw: GACGAGCACGAACCCTCGAT;<br>Rv: TGCAGCTTCCCTGCACAGA
CDT1	ENSG00000167513	Fw: AAGGATCCCGCCTACCAGCGCTTCC;<br>Rv: CCAAGCTTGAAGGTGGGGACACTG
HAT1	ENSG00000128708	Fw: TGATGAAAGATGGCACTACTTTCTAGT;<br>Rv: AGCCTACGGTCGCAAAGAG
RAN	ENSG00000132341	Fw: GGATATTAAGGACAGGAAAGTG;<br>Rv: TGGGTCCATGACAACTTCTG
DYNC1H1	ENSG00000197102	Fw: GACGTCGGTGATGAAGGAGAAG;<br>Rv: TCTGCATCAATCACGGGAGTAC
MYC	ENSG00000136997	Fw: TCAAGAGGCGAACACACAAC;<br>Rv: GGCCTTTTCATTGTTTTCCA
TRIB3	ENSG00000101255	Fw: GCCCTGCACTGCCCTACAG:<br>Rv: GGTACCAGCCAGGACCTCAGT
RRM2	ENSG00000171848	Fw: GCAGCAAGCGATGGCATAGT;<br>Rv: GGGCTTCTGTAATCTGAACTTC
MCM3	ENSG00000112118	Fw: GAGTGAATCCAGGTTGAAGG;<br>Rv: GATTCTGTGAGGCGATTCAT
CBS	ENSG00000160200	Fw: ACATGCTCTCGTCCCTGCTT;<br>Rv: GTGAGGCGGATCTGTTTGAACT
PEG10	ENSG00000242265	Fw: AACAACAACAACAACTCCAAGC;<br>Rv: TCTGCACCTGGCTCTGCAG
E2F1	ENSG00000101412	Fw: CCCAACTCCCTCTACCCTTGA;<br>Rv: TCTGTCTCCCTCCCTCACTTTC
SLC2A1	ENSG00000117394	Fw: GATTGGCTCCTTCTCTGTGG;<br>Rv: TCAAAGGACTTGCCCAGTTT
STMN1	ENSG00000117632	Fw: AAGGATCTTTCCCTGGAGGA;<br>Rv: TGTGCCTCTCGGTTCTCTTT
PBK	ENSG00000168078	Fw: CCTTTGGCCTTACTTTGTG;<br>Rv: ACGATCTTTAGGGTCTTCAT
DDIT3	ENSG00000175197	Fw: AGAGCCCTCACTCTCCAGATTCCA;<br>Rv: TCTGTTTCCGTTTCCTGGTTCTCC
ITGA6	ENSG026407R291409	Fw: GCTGGTTATAATCCTTCAATATCAATTG;<br>Rv: TTGGGCTCAGAACCTTGGTTT
CLIC4	ENSG00000169504	Fw: AGCAGAAGCAGCAGCAG;<br>Rv: TATACCTTGTCTATCCTTGATCCTA
NFIB	ENSG00000147862	Fw: CCACCTTCACCGTTGCCATTTC;<br>Rv: CCAGGACTGGCTGGTTTGTGGA
TAF15	ENSG00000172660	Fw: GCCTGGCTTTCGTATTCGTTG;<br>Rv: CGACGGTCATCTTGTGTGTGG
MTHFD2	ENSG026407R265911	Fw: CGCGGCAGTTCGAAATGAAGCTG;<br>Rv: ATCAGATTTGGAATACCTGCAGC
MIA	ENSG00000150526	Fw: CATGCATGCGGTCCTATGCCCAAGCTG;<br>Rv: GATAAGCTTTCACTGGCAGTAGAA
PDK1	ENSG00000152256	Fw: GCACGGCAAGGGCATCATTC;<br>Rv: CGACATAGCCGGCAGGTAAGC
18rs		Fw: GGAGTATGGTTGCAAAGCTGA;<br>Rv: ATCTGTCAATCCTGTCCGTGT

Supplementary Table 2: Primer sequences used for pyrosequencing and shRNA sequences used for gene knockdowns

Primer sequences used for pyrosequencing			
**Gene**	**Primer sequences for pyrosequencing**	**Annealing Tm[°C]**	**Amplicon length [bp]**
STMN1	FW: 5'-GATGATAGGGGAGGAAGAGTAATA-3'<br>RV: BIO-5'-AACCATATTCATTTCATTTATTTTTTTCC-3'<br>SEQ1: 5'-TTTTAGAGTAGTATTGGGTT-3'<br>SEQ2: 5'-GGTATAAAGGAAATTGGTTTG-3'	58	267
TAF15	FW:5'-GGTTTTGTAGGATAGAAATGTGTGATTA-3'<br>RV:BIO-5'-AACTTTATCAAACTTCATCCCAAATTAC-3'<br>SEQ:5'-ATAGAAATGTGTGATTAAAATGA-3'	60	302
CLIC4	FW: BIO-5'-ATGGTTTAAGTTTTTATTTAAGGGGAGTT-3'<br>RV: 5'-AAACCCCTAAACCTCAATTTCATT-3'<br>SEQ:5'-AAATATAACATCATATTCTACTCTC-3'	61	239
NFIB	FW:5'-GGGTTGTTGGGATTAGGAAGATA-3'<br>RV:BIO-5'-TAACCCTATCACCTCATTTCTTTCAT-3'<br>SEQ:5'-GGGATTAGGAAGATAGGTA-3'	61	241
DYNC1H1	FW:5'-AGTGAAGGTGTTATTTTTGGTTTAATT-3'<br>RV:BIO-5'-ATAACACACAACCAAAAAAACTTTA-3'<br>SEQ:5'-ATTTTTGGTTTAATTTTATATTGTT-3'	60	338
PEG10	FW: BIO-5'-GATATTGAGGAGTTTAGATGGGAATT<br>RV: 5'-CACAAAAACCTTACTTCCCTTACA<br>SEQ:5'-ATTCTAATAAAAAAAAATACTAAAT	59	265
RAN	FW:5'-TTGAGGGTTTTGGGGGAGTT-3'<br>RV:BIO-5'-TCAACTTCCCAACCCCCTAT-3'<br>SEQ:5'-GGTTTTTTTTTTATGGTGGA-3'	60	246

List of shRNA sequences used in the study (Open Biosystems)		
**Catalog #**	**#**	**Mature Antisense**
ShScr [RHS4346]		(non-silencing GIPZ lentiviral shRNAmir control; contains no homology to known mammalian genes)
STMN1	V2LHS_62940	5'-TTATTAACCATTCAAGTCC-3'
	V2LHS_263357	5'-TACAGTACTAGCCATTAAC-3'
	V3LHS_383501	5'-TTCTTCTGAATTTCCTCCA-3'
	V3LHS_383503	5'-ATCCTTCTCTCGCAAACGT-3'
	V3LHS_383504	5'-TCTCTTCTATTGCCTTCTG-3'
	V3LHS_383505	5'-TTATTAGCTTCCATTTTGT-3'
TAF15	V2LHS_172493	5'-TTCCGCATGACGGATTAGG-3'
	V2LHS_172494	5'-ACAGTCATCAGTATGGTCG-3'
	V3LHS_334750	5'-TGATCTGTAGACACACCCT-3'
	V3LHS_334751	5'-TGTTGACCATAGTCTGGCT-3'
	V3LHS_334754	5'-TAGGTAGAATAACTTTGCT-3'
	V3LHS_637402	5'-TGACTATAGGAATCATGCT-3'

Supplementary Table 3: Summary of RNA sequencing raw data

Sample	No.	Reads	%Duplicate
HepG2.ctrl	1	29,149,120	20.90
	2	29,039,184	20.95
HepG2.SAM	1	32,471,287	20.06
	2	32,326,850	20.11
Skhep1.ctrl	1	33,250,094	19.59
	2	33,092,614	19.64
Skhep1.SAM	1	30,574,959	17.34
	2	30,420,913	17.37
NorHep.ctrl	1	29,772,669	18.05
	2	29,097,853	20.18
NorHep.SAM	1	29,244,839	20.13
	2	29,621,328	18.10

Supplementary Table 4: Differentially expressed genes in SAM treated cells versus untreated cells

Cell line	Up-regulated	Down-regulated
HepG2	3320	3342
Skhep1	2689	2860
NorHep	2587	2376

Supplementary Table 5: List of differentially regulated genes between untreated cancer cell lines and normal liver cell line (sk_nor) which were significantly affected by SAM treatment (sk_SAM) (fc=fold change) (see table_s5 excel file)

See Supplementary File 1

Supplementary Table 6: List of genes related to the IPA-defined bioFunction liver "disease" pathway (liver cancer, liver tumor, hepatobiliary system cancer, proliferation of tumor cell lines and invasion of tumor cell lines) that are differentially expressed in HepG2 and SKhep1 and are reversed by SAM (see table_s6 excel file)

See Supplementary File 2

Supplementary Table 7: Summary of capture bisulfite sequencing raw data

Sample	Reads	%Duplicate
HepG2.ctrl	55,547,269	8.7
HepG2.SAM	72,869,382	10.2
Skhep1.ctrl	71,936,330	9.8
Skhep1.SAM	50,900476	8.1
NorHep.ctrl	61,618,983	8.9
NorHep.SAM	65,662,044	9.7

Supplementary Table 8: Summary of the frequency of distribution of methylation levels in the three untreated and SAM treated cell lines in each annotated genomic feature (see table_s8 excel file)

See Supplementary File 3

Supplementary Table 9: Summary of number of CpG and non-CpG sites that change their state of methylation in response to SAM treatment in each cell line (%|meth.diff|>=15%, q value<0.05)

CpG type	CpGs		Non-CpGs	
**difference**	**Hypermethylation**	**Hypomethylation**	**Hypermethylation**	**Hypomethylation**
HepG2	441017	424292	32545	52543
Skhep1	462021	442113	51868	29061
NorHep	315914	323600	36379	39679

Supplementary Table 10: List of genes that are both hypermethylated (in their promoters) and down-regulated or both hypomethylated (in their promoters) and up-regulated in HepG2 (HepC_norC) and Skhep1 (SKC-norC) cancer cell lines in comparison with NorHep (mdiff=methylation difference; logFC=log fold change in expression) (see table_s10 excel file)

See Supplementary File 4

Supplementary Table 11: List of genes that are both hypomethylated (HepC/SkC-norC.mdiff) (in their promoters) and are up-regulated in HepG2 (HepC_norC.logFC) and Skhep1 (SkC_norC.logFC) cells relative to NorHep whose state of methylation (SAM_HepC/SkC.mdiff) and expression (SAM_HepC/SkC.logFC) is reversed by SAM treatment (see table_s11 excel file)

See Supplementary File 5

Supplementary Table 12: List of the genes that are both hypomethylated (in their promoters) (HepC/SkC-norC.mdiff) and upregulated in their expression (HepC/SkC-norC.logFC) in both HepG2 and SKHep1 versus NorHep whose state of methylation (SAM_HepC/SkC.mdiff) and expression (SAM_HepC/SkC.logFC) was reversed by SAM (in at least one cancer cell line) that are involved in the IPA biofunctions of cell proliferation and cell invasion (see table_s12 excel file). (common proli_tumor=reversed in both tumor cell lines; unique=reversed in one of the cell lines)

See Supplementary File 6
